# Using single-index ODEs to study dynamic gene regulatory network

**DOI:** 10.1371/journal.pone.0192833

**Published:** 2018-02-23

**Authors:** Qi Zhang, Yao Yu, Jun Zhang, Hua Liang

**Affiliations:** 1 Department of Statistics, Qingdao University, Qingdao, China; 2 Department of Biostatistics and Computational Biology, University of Rochester School of Medicine and Dentistry, Rochester, New York, United States of America; 3 Institute of Statistical Sciences at Shenzhen University, Shenzhen University, Shenzhen, China; 4 Department of Statistics, George Washington University, Washington, D.C., United States of America; University of Toronto, CANADA

## Abstract

With the development of biotechnology, high-throughput studies on protein-protein, protein-gene, and gene-gene interactions become possible and attract remarkable attention. To explore the interactions in dynamic gene regulatory networks, we propose a single-index ordinary differential equation (ODE) model and develop a variable selection procedure. We employ the smoothly clipped absolute deviation penalty (SCAD) penalized function for variable selection. We analyze a yeast cell cycle gene expression data set to illustrate the usefulness of the single-index ODE model. In real data analysis, we group genes into functional modules using the smoothing spline clustering approach. We estimate state functions and their first derivatives for functional modules using penalized spline-based nonparametric mixed-effects models and the spline method. We substitute the estimates into the single-index ODE models, and then use the penalized profile least-squares procedure to identify network structures among the models. The results indicate that our model fits the data better than linear ODE models and our variable selection procedure identifies the interactions that may be missed by linear ODE models but confirmed in biological studies. In addition, Monte Carlo simulation studies are used to evaluate and compare the methods.

## Introduction

Gene regulatory networks (GRN) are complex and dynamic systems in nature. They are composed of genes that interact with each other and with other substances inside cells, such as RNAs and proteins. Over the past few decades, a variety of methods have been proposed to model GRN. Commonly used models include information theory models, Boolean networks, ordinary differential equation (ODE) models, and Bayesian networks [1]. Information theory models [2–4] construct network architecture on correlation coefficients. Such models are simple and have a low computation cost, but cannot take into account the dynamic processes and situations when multiple genes participate in regulations. Boolean networks [5–7] are discrete dynamic networks and easy to understand, but have limitations because their networks’ nodes are binary states: “off” or “on”. Due to these simplifying assumptions, the study of kinetic gene regulation is still challenging because of the complexity of the biological process [8].

The Bayesian networks [9–12] integrate biological knowledge and measurements to infer network structures. But the estimated results obtained from Bayesian networks depend on the quality and completeness of prior knowledge. As pointed out by [13], the existing ODE models and associated methods used to study GRN are flexible but are limited to small scale gene expression levels. ODE models describe the dynamic behaviors of GRN in a quantitative manner and represent gene expression level changes by functions of gene expression levels:

$$\begin{array}{c} \frac{dX_{k}\left(t\right)}{dt}=F\left(t,X\left(t\right),\theta \right),\;k=1,\ldots ,p, \end{array}$$ (1)

where **X**(*t*) = (*X*_1_(*t*), ⋯, *X*_p_(*t*))^T^ represents gene expression levels at the time *t* of the *p* genes; *F*(⋅, ⋅, ⋅) is a function which can be linear or nonlinear; and ***θ*** is an unknown parameter vector which quantifies the regulations or interactions among the genes in GRN.

Once we can determine **X**(*t*), the gene expression levels which should be included in the ODE model (1), we can infer the interactions within a dynamic GRN. This motivates us to use appropriate models and to develop associated techniques in order to construct dynamic GRN for time course gene expression data. Within a dynamic GRN, the majority of the genes are not significantly relevant to each other. The precision of parameter estimation, model interpretability, and the accuracy of forecasting will be reduced when irrelevant genes are included in models [14]. Thus, those irrelevant genes should be excluded from the final model. However, variable selection for ODE models using traditional statistical methods is important but challenging, especially when it comes to dynamic GRN. The difficulties arise from two aspects: one is the collinearity among genes, i.e., genes sharing same “pathway” are highly correlated in expressions; the other is the high-dimensional feature of GRN, i.e., a large-scale GRN involves hundreds or even thousands of genes. When the number (*n*) of measurements for individual genes is much smaller than the number (*p*) of genes, traditional statistical methods face significant challenges in developing statistical procedures and deriving theory [15].

Pioneering research has investigated gene regulatory networks using variable selection techniques. For example, [13] proposed linear ODE models: *dX_k_*(*t*)/*dt* = *γ*^T^**X**(*t*) and developed a variable selection procedure based on SCAD penalty. [13] further employed their method to construct a module-based dynamic network. However a linear ODE model has many limitations and is unable to capture certain patterns. In reality, the first derivatives of the gene expression profiles (the time-related changes of a gene expression) can be quantified as a function of gene expression levels of all related genes. The link functions that quantify the regulatory effects of genes on the first derivatives may be nonlinear. In other words, systems of cellular regulations may be nonlinear [1, 16]. Due to the limitations of linear ODE models, developing a flexible modeling approach to explore the interactions among genes has become necessary. When the linear assumption cannot be satisfied, it is natural to consider a single-index model, *E*(*Y*|*X*) = *η*(*X*^*T*^ *β*) with *η* being an *unknown* differentiable function and *β* an unknown parameter to be estimated. Single-index models have many advantages, such as being able to model the curvature of a smooth curve and circumventing the so-called “curse of dimensionality”. More discussions about the usefulness of single-index models are provided in [17]. A nonlinear ODE model (given the function *η*) may suffer from misspecification and “the curse of dimensionality”, whereas single-index ODE models can avoid these two problems and are more flexible, and the index parameter (*β*) can be estimated with the root—*n* convergence rate though the link function is unknown. More importantly, single index ODE models allow the predictors to have interactions, which is common in characterizing gene-gene regulation.

Various methods have been proposed to estimate regression coefficients for single-index models. See [18–23] for parameter estimators. In addition, much research has been done on variable selection for single-index models. For example, [24] developed a variable selection method based on sliced inverse regression. [25] proposed a leave-*m*-out cross-validation method to select variables in a single-index model. [26] proposed semiparametrically efficient profile least-squares estimators for parameter estimation, and employed the SCAD approach to simultaneously select variables and estimate regression coefficients. [27] studied estimation and variable selection coupling with dimension reduction procedures.

Although parameter estimation and variable selection for single-index models have gained fruitful results, to the best of our knowledge, no method that couples single-index models with ODE to study dynamic GRN is available. In this paper, we propose a single-index ODE model to study dynamic GRN with the aim of overcoming the inadequacy of linear ODE models. This model can be written as

$$\begin{array}{c} \frac{dX_{k}\left(t\right)}{dt}=\eta_{k}(X{\left(t\right)}^{\text{T}}\beta_{0}^{\left[k\right]})+\varepsilon ,k=1,\ldots ,p, \end{array}$$ (2)

where *η*_*k*_(⋅) is an unknown differentiable function; $\beta_{0}^{\left[k\right]}$ is a parameter vector with $∥\beta_{0}^{\left[k\right]}∥=1$, and the first element of $\beta_{0}^{\left[k\right]}$ is positive (for identifiability), where ∥ ⋅ ∥ denotes the Euclidean norm. **X**(*t*) = (*x*_1_(*t*), ⋯, *x*_p_(*t*))^T^ are state functions. Here **X**(*t*) can be gene-expressing levels of genes or population mean curves for functional modules. To study the interactions within dynamic GRN, one needs to identify the relevant **X**(*t*) for ODE models, that is $\beta_{0}^{\left[k\right]}\neq 0$. We therefore apply the penalized least-squares approach for this aspect and for estimating dynamic parameters $\beta_{0}^{\left[k\right]}$.

**Fig 1:**
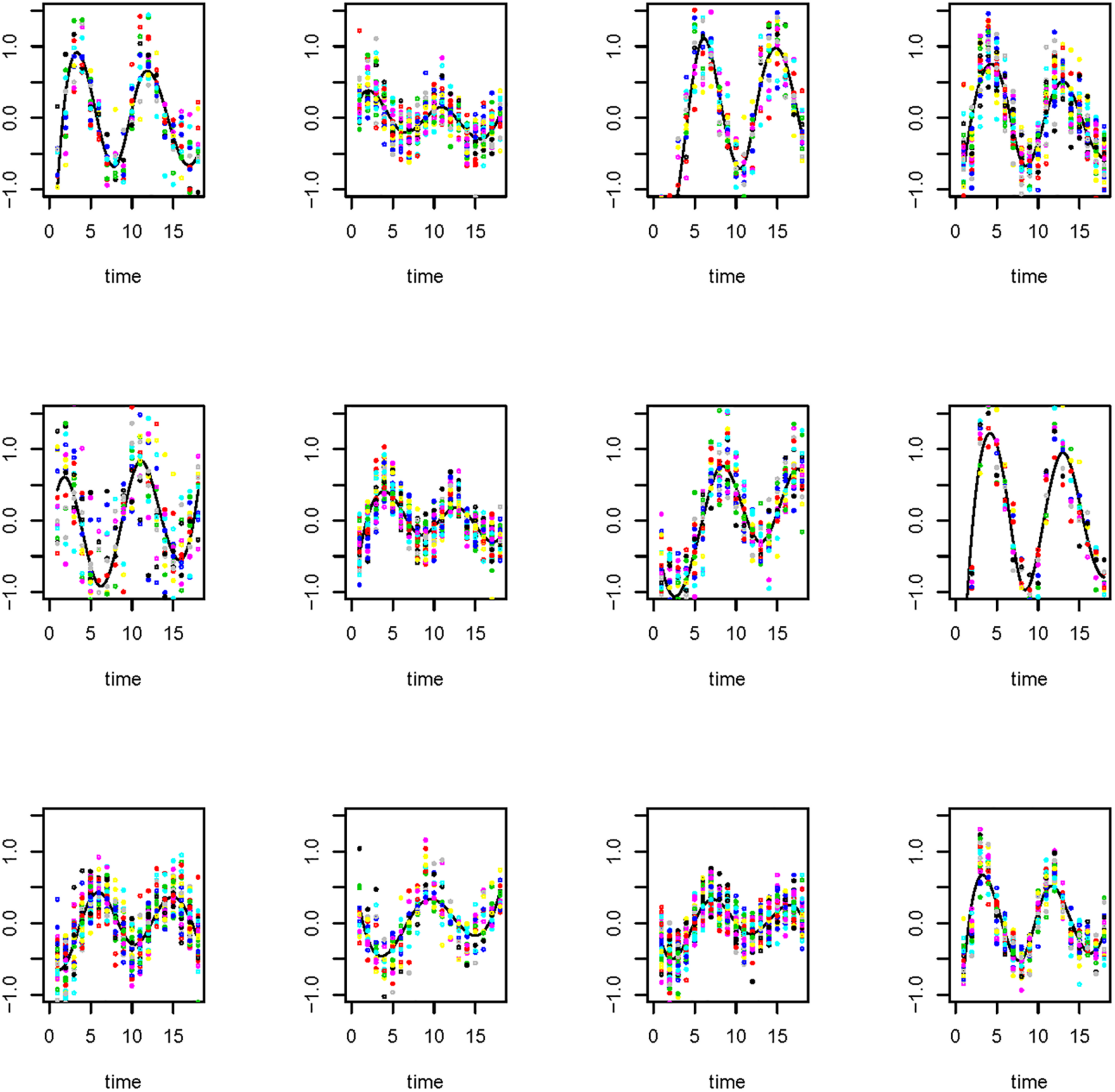
The scatterplot of gene expressions against time (the same color for each individual gene in a module) and the population mean curve (solid line) of 12 modules for the time course yeast cell data set.

We will apply the mixed-effects nonparametric model with a mixture distribution framework to cluster the genes into functional modules in the first step. This clustering approach allows us to build the module-based dynamic network and identify the interesting functional modules. These interesting modules may play important roles in ‘dynamic’ regulations. Although these interesting modules may contain many genes with heterogeneous functions, it can allow scientists to focus on the genes in each module for further investigations. As shown in Fig 1 (below), most gene expression levels can be grouped in several clusters. In each cluster, these expression levels share a similar pattern. The genes in a cluster (represented by a node) may play a common function in biological procession. Such a network can single out regulator-regulator interactions which are helpful to avoid tedious experiments and to speed biological studies.

In Section of Methods, we briefly describe the procedure for GRN construction with details for penalized profile least-squares (PPrLS) estimation and variable selection. In Section of Numerical Results, we construct a module-based GRN structure by using PPrLS estimator for the yeast cell cycle gene expression data with additional results (S1 and S2 Tables), and conduct Monte Carlo simulation studies to evaluate the performance of the proposed procedure. The simulation settings were designed to mimic the gene expression patterns from the real data example. In Section of Discussions, we conclude the article with a brief discussion. All theory and associated technical details are given in the supporting materials (S1–S7 Files).

## Methods

Time-course gene expressions are synchronized to many ongoing biological processes such as tissue repair, cell differentiation, or cell cycles [28, 29]. Through understanding the genes underlying the cell cycles, we can study the mechanisms of many diseases at a molecular level and in turn provide potential drug targets for treating those diseases. From the end of the last century, the identification of cell cycles associated genes has attracted considerable attention in biological study. For example, [28, 30] performed genome-wide transcriptional analysis of the cell cycle process of yeast using microarrays and identified about 800 cell-cycle-regulated genes. GRN include genes, the products of genes, and the interactions among them, which together affect many cellular processes. To understand the dynamic mechanism of cellular processes, modeling and analysis of dynamic gene regulatory networks using time-course gene expression data has attracted much attention. We model dynamic network for functional modules based on observed time course gene expression levels in three steps.

**Step 1.** Group genes into functional modules using the smoothing spline clustering (SSC) approach [31–33]. Final number of clusters was selected by the Bayesian information criterion (BIC), and penalty parameters were determined by the leave-one-out cross-validation procedure (GCV);
**Step 2.** Estimate state function *X*(*t*) and first derivative *X*′(*t*) for each functional module using nonparametric mixed-effect models (NPME) and the spline method respectively;
**Step 3.** Select modules and estimate dynamic parameters using the PPrLS procedure given below, for which tuning parameter was selected by the BIC, and bandwidths were determined by the GCV.

We now describe the details for these three steps.

### Step 1—Clustering process

We assume the time-course gene expression levels for gene *i* can be represented by a smooth function of time and follow a mixture Gaussian distribution:

$$\begin{array}{c} g_{i}\left(t\right)∼p_{1}N\left(\mu_{1},\Sigma_{1}\right)+p_{2}N\left(\mu_{2},\Sigma_{2}\right)+\cdots +p_{p}N\left(\mu_{p},\Sigma_{p}\right), \end{array}$$ (3)

where *p*_*k*_, *k* = 1, ⋯, *p* are the probability that gene *i* belongs to cluster *k*; *μ*_*k*_, *k* = 1, ⋯, *p* and Σ_*k*_, *k* = 1, ⋯, *p* are the vector representations of the mean curve and variances components for each cluster respectively. The gene expression levels for individual genes are assumed to follow an overall mean curve (fixed-effect) while having a gene-specific shift (random-effect). Therefore nonparametric mixed-effect model can be constructed by fitting the time-course gene expressions for each gene to a function over time by using the smoothing spline method. Through maximizing the penalized log-likelihood, the SSC procedure estimates the probabilities *p*_*k*_. The means *μ*_*k*_ and variances component Σ_*k*_ can be estimated as by-products also. More details about the SSC procedure are available in [32] and [33].

### Step 2—Applications of nonparametric mixed-effect models

After grouping genes into functional modules, we apply NPME models to estimate the state function *X*(*t*) and its first derivative *X*′(*t*) for each functional module. For notation simplicity, we consider the estimation of the state function and its first derivative for module 1 (*k* = 1) and denote them by *X*(*t*) and *X*′(*t*) respectively. Suppose the number of genes in module 1 is *m*, and the number of measurements collected from each gene is *m*_*i*_. The NPME model can be described as

$$\begin{array}{c} g_{i}(t=X\left(t\right)+v_{i}\left(t\right)+\varepsilon_{i}\left(t\right),\;i=1,\cdots ,m, \end{array}$$ (4)

where *g*_*i*_(*t*) is the observed gene expression level for the *i*^*th*^ gene; *X*(*t*) presents the fixed-effect or population curve which reflects an overall time-related trend of the gene expression level for module 1; *v*_*i*_(*t*) describe individual curve variations; *ε*_*i*_(*t*) are measurement errors; and *v*_*i*_(*t*) and *ε*_*i*_(*t*) are assumed to be independent.

We can combine the penalized spline [34–36] with the linear mixed-effects (LME) modeling framework [37] to approximate *X*(*t*). For presentation completeness, we briefly summarize the estimation procedure. We first approximate X(t) and *v*_*i*_(*t*) by $\tilde{X}\left(t\right)$ and ${\tilde{v}}_{i}\left(t\right)$, respectively, which are expressed as:

$$\begin{array}{c} \tilde{X}\left(t\right)=\sum_{r=0}^{l}\alpha_{r}t^{r}+\sum_{r=1}^{R}u_{r}{\left(t-\zeta_{r}\right)}_{+}^{l},\;\text{and}\;\;{\tilde{v}}_{i}\left(t\right)=\sum_{r=0}^{l}b_{ir}t^{r}+\sum_{r=1}^{R}w_{ir}{\left(t-\zeta_{r}\right)}_{+}^{l}, \end{array}$$ (5)

where *l* ≥ 1 is an integer, *ζ*_1_ < ⋯ < *ζ*_*R*_ are fixed knots, *u*_*r*_(*t* − *ζ*_*r*_)_+_ = max(0, *t* − *ζ*_*r*_), ***α*** = (*α*_1_, ⋯, *α*_*l*_), **u** = (*u*_1_, ⋯, *u*_*R*_), **b**_*i*_ = (*b*_*i*0_, ⋯, *b*_*il*_), and **w**_*i*_ = (*w*_*i*0_, ⋯, *w_iR_*). Let

$$S_{i}=(\begin{array}{cccc} 1 & t_{i1} & \cdots & t_{i1}^{l}\\ 1 & t_{i2} & \cdots & t_{i2}^{l}\\ \vdots & \vdots & \ddots & \vdots \\ 1 & t_{im_{i}} & \cdots & t_{im_{i}}^{l} \end{array}),\;\text{and}\;Z_{i}=(\begin{array}{ccc} {\left(t_{i1}-\zeta_{1}\right)}_{+}^{l} & \cdots & {\left(t_{i1}-\zeta_{R}\right)}_{+}^{l}\\ {\left(t_{i2}-\zeta_{1}\right)}_{+}^{l} & \cdots & {\left(t_{i1}-\zeta_{R}\right)}_{+}^{l}\\ \vdots & \ddots & \vdots \\ {\left(t_{im_{i}}-\zeta_{1}\right)}_{+}^{l} & \cdots & {\left(t_{im_{i}}-\zeta_{R}\right)}_{+}^{l} \end{array}).$$

The approximation of model (4) can be expressed as

$$\begin{array}{c} g=S\alpha +\Lambda b+Zu+\Gamma w+\varepsilon , \end{array}$$ (6)

where $S={\left(S_{1}^{\text{T}},\cdots ,S_{m}^{\text{T}}\right)}^{\text{T}}$, $g={\left(g_{1}^{\text{T}},\cdots ,g_{m}^{\text{T}}\right)}^{\text{T}}$, $\Lambda =\text{diag}(S_{1}^{\text{T}},\cdots ,S_{m}^{\text{T}})$, $Z={\left(Z_{1}^{\text{T}},\cdots ,Z_{m}^{\text{T}}\right)}^{\text{T}}$, $\Gamma =\text{diag}(Z_{1}^{\text{T}},\cdots ,Z_{m}^{\text{T}})$, $b={\left(b_{1}^{\text{T}},\cdots ,b_{m}^{\text{T}}\right)}^{\text{T}}$, and $w={\left(w_{1}^{\text{T}},\cdots ,w_{m}^{\text{T}}\right)}^{\text{T}}$. Model (6) is a standard LME model. As a result, ***α***, **b**, **u** and **w** can be estimated by using the function lme (available in the R package nlme). Substituting the estimated $\hat{\alpha }$ and $\hat{u}$ in Eq (5), we estimate the *X*(*t*) for module 1. After estimating **X**(*t*), we apply the spline method (available in the R package splines) to estimate the first derivative of $\hat{X}\left(t\right)$. The detailed estimation procedure is referred to [38] and [39].

### Step 3—Estimation procedure based on the penalized profile least-squares approach

Suppose a genome-wide time course gene expression levels were clustered into *p* modules; *X*_*j*_(*t*), *j* = 1, ⋯, *p* are the population mean curves estimated by NPME models; and ${\hat{X}}_{k}^{\prime }\left(t\right)$ are the estimates of the first derivative *dX*_*k*_(*t*)/*dt* for the *k*-th module. Substituting *X*_*j*_(*t*), *j* = 1, ⋯, *p* and the first derivative ${\hat{X}}_{k}^{\prime }\left(t\right)$ for the *k*-th module in model (2), we obtain a single-index ODE model for the *k*-th functional module which can be written as

$$\begin{array}{c} Y_{k}\left(t\right)=\eta_{k}(X{\left(t\right)}^{\text{T}}\beta_{0}^{\left[k\right]})+\varepsilon ,\;k=1,\cdots ,p, \end{array}$$ (7)

where *η*_*k*_ is an unknown differentiable function, $Y_{k}\left(t\right)={\hat{X}}_{k}^{\prime }\left(t\right)$, **X**(*t*) = (*X*_1_(*t*), …, *X*_p_(*t*))^T^, $\beta_{0}^{\left[k\right]}={\left(\beta_{01}^{\left[k\right]},\cdots ,\beta_{0p}^{\left[k\right]}\right)}^{\text{T}}$, and *ε* is the sum of numerical errors due to integration and estimation. This complexity *ε* makes it challenging to study the properties of the proposed estimator for ***β**′s*, For simplicity, we adopt an additive error model used in the literature [13, 40, 41]. Once the **X**(*t*) can be identified, we construct a module-based network. Here we develop the variable (population mean of functional modules) selection and estimation procedure for model (7) based on the penalized profile least-squares approach as follows.

Selecting variables by penalized least squares has been widely studied in literature. See, for example, the least absolute shrinkage and selection operator (LASSO) [42], the smoothly clipped absolute deviation (SCAD) approach [43], the adaptive lasso estimator [44], the elastic-net estimator [45] and the adaptive elastic-net estimator [46]. However, the variable selection problem for single-index ODE models has not been addressed in the literature. In this paper, we extend the approach proposed by [26] to the single-index ODE model (7).

Let *p* be the number of all modules; *X_i_* = (*X*_1_(*t_i_*), …, *X_p_*(*t_i_*))^T^, *i* = 1, …, *N*, $Y_{i}={\hat{X}}_{k}^{\prime }\left(t_{i}\right)$ be the vector representations of the mean curves of *p* function modules and the estimates of the first derivative *dX*_*k*_(*t*)/*dt* for the *k*-th module. Assume the functional data of the *k*th module follows the single-index ODE model

$$\begin{array}{c} Y_{i}=\eta_{k}(X_{i}^{\text{T}}\beta^{\left[k\right]})+\varepsilon_{i},\;k=1,\cdots ,p, \end{array}$$ (8)

Let $\Lambda_{i}=X_{i}^{\text{T}}\beta^{\left[k\right]}$. *η*_*k*_(*u*) can be estimated utilizing the local linear regression method [47], i.e., minimizing

$$\begin{array}{c} \sum_{i=1}^{N}{\left\{a_{k}+b_{k}\left(\Lambda_{i}-u\right)-Y_{i}\right\}}^{2}K_{h}\left(\Lambda_{i}-u\right), \end{array}$$ (9)

with respect to *a*_*k*_ and *b*_*k*_, where *K*_*h*_(⋅) = *K*(⋅/*h*)/*h*, *K*(⋅) is a kernel function and *h* is a bandwidth. We can then obtain

$$\begin{array}{c} {\hat{\eta }}_{k}\left(u,\beta \right)={\hat{a}}_{k}=\frac{K_{20}\left(u,\beta \right)K_{01}\left(u,\beta \right)-K_{10}\left(u,\beta \right)K_{11}\left(u,\beta \right)}{K_{00}\left(u,\beta \right)K_{20}\left(u,\beta \right)-K_{10}^{2}\left(u,\beta \right)}, \end{array}$$ (10)

where $K_{jl}\left(u,\beta \right)=\sum_{i=1}^{N}K_{h}\left(X_{i}^{\text{T}}\beta^{\left[k\right]}-u\right){\left(X_{i}^{\text{T}}\beta^{\left[k\right]}-u\right)}^{j}Y_{i}^{l},$ for *j* = 0, 1, 2 and *l* = 0, 1, 2. Consequently, the profile least squares function can be proposed as a function of ***β***^[*k*]^

$$\begin{array}{c} Q\left(\beta^{\left[k\right]}\right)=\sum_{i=1}^{N}{\left\{Y_{i}-{\hat{\eta }}_{k}\left(X_{i}^{\text{T}}\beta^{\left[k\right]}\right)\right\}}^{2}. \end{array}$$ (11)

The above estimation procedure can be used when the true model is known a priori. Because we wish to identify GRN structure and enhance the predictive power of a proposed model, we apply the penalized least-squares approach to simultaneously select modules and estimate parameters. Define a penalized profile least-squares (PPrLS) function

$$\begin{array}{c} L_{P}(\beta^{\left[k\right]})=\frac{1}{2}Q(\beta^{\left[k\right]})+N\sum_{j=1}^{p}p_{\lambda^{\left[k\right]}}(|\beta_{j}^{\left[k\right]}|), \end{array}$$ (12)

where *p*_*λ*^[*k*]^_(⋅) is a penalty function with a regularization parameter *λ*^[*k*]^. The PPrLS estimator of ***β***^[*k*]^ is the minimizer of Eq (12); i.e.,

$$\begin{array}{c} {\hat{\beta }}^{\left[k\right]}=\text{argmin}L_{P}(\beta^{\left[k\right]}). \end{array}$$ (13)

For a given tuning parameter *λ*^[*k*]^, we can estimate ***β***^[*k*]^ by minimizing $L_{P}(\beta^{\left[k\right]})$ with respect to ***β***^[*k*]^. By determining non-zero ***β***^[*k*]^, we identify the modules having impacts on the *k*th module and therefore construct GRN.

There are various penalty functions in the literature of variable selection for semiparametric models. Considering the SCAD method has many good theoretical properties, we adopt the SCAD penalty function [43], and adopt BIC selector proposed by [48] to choose the regularization parameters *λ*^[*k*]^ by minimizing the following objective function:

$$\begin{array}{c} \text{BIC}\left(\lambda^{\left[k\right]}\right)=\log\left\{\text{MSE}\left(\lambda^{\left[k\right]}\right)\right\}+\left\{\log\left(N\right)/N\right\}{\text{DF}}_{\lambda^{\left[k\right]}}, \end{array}$$ (14)

where $\text{MSE}\left(\lambda^{\left[k\right]}\right)=N^{-1}\sum_{i=1}^{N}{\left\{Y_{i}-{\hat{\eta }}_{k}\left(X_{i}^{\text{T}}{\hat{\beta }}_{\lambda^{\left[k\right]}}^{\left[k\right]}\right)\right\}}^{2}$ and DF_*λ*^[*k*]^_ is the number of nonzero coefficients of ${\hat{\beta }}_{\lambda^{\left[k\right]}}^{\left[k\right]}$, the PPrLS obtained from (12) for each *λ*^[*k*]^.

**Remark**. Although the proposed method needs three steps to implement and its computational cost is high, compared to the existing methods, its gain in computational efficiency is significant. Most of dynamic network models such as dynamic Bayesian networks and random graph models require extensive computations for posterior inference. As a result, Bayesian based methods allow one to deal with only small networks. The proposed method can avoid numerically solving the differential equations directly, and does not need the initial or boundary conditions of the state variables. The method also incorporate the high-dimensional ODEs to allow us to perform variable selection and parameter estimation for one equation. These good features gain computational efficiency.

## Numerical results

### Real data analysis

We used the procedure introduced in Section of Methods to analyze a time-course yeast cell cycle gene expression data set. These 297 genes were identified as expressions across 18 time points during approximate two cell cycles; i.e., each gene has 18 time-related observations [49].

We implemented Step 1 using the MFDA function (available in the R package MFDA), and identified 12 functional modules. The population mean curves for the functional modules are given in Fig 1. We can see that for each functional module, the genes included share a similar pattern. These time-related patterns show two cell cycles (Fig 1). The number of genes included in each functional module ranges from 9 to 53.

In order to construct a functional landscape of the genome-wide regulatory network through identifying interactions among modules, we used the Database for Annotation, Visualization and Integrated Discovery [50, 51] to identify enriched functional annotations in Gene ontology and Kyoto Encyclopedia of Genes and Genomes pathways for each functional module. A modified Fisher exact test was used to test the null hypothesis that a certain function is not over-represented in the module compared to the background population. Due to space limitation, we displayed part of the selected functional annotations in Table 1. All enriched functional annotations were provided in S1 Table.

**Table 1:** The inward and outward regulations in the module-based regulatory network and RSS based on the linear ODE (L-ODE) and the single-index ODE (Si-ODE).

Module	Selected function annotation and associated p-values (in parentheses)	Outward influence modules		Inward influence modules		RSS	
		L-ODE	Si-ODE	L-ODE	Si-ODE	L-ODE	Si-ODE
module1 (12)	DNA replication (0.013), regulation of RNA metabolic process (0.014), meiosis (0.021)	3, 5, 7, 8	1, 3, 7, 8, 9, 12	2, 6, 9, 12	1, 2, 3, 4, 5, 8, 9, 10, 11	3.07E-03	1.41E-04
module2 (30)	cellular carbohydrate biosynthetic process (0.006),	1, 7, 9	1, 2, 7, 8, 9, 12	7	2, 4, 5, 8, 9, 10, 12	5.20E-04	3.38E-05
module3 (15)	protein—DNA complex assembly (< 0.001), DNA packaging (< 0.001)	3, 7, 8, 10	1, 7, 8, 12	1, 3, 4, 5, 6, 7, 8, 12	1, 4, 5, 6, 7, 8, 10, 11, 12	3.37E-03	3.58E-03
module4 (32)	DNA metabolic process (< 0.001), DNA replication (< 0.001), DNA repair (< 0.001), cell-division cycle (0.025)	3, 5, 7, 8	1, 2, 3, 7, 8, 9, 11, 12	NA	6, 8, 9, 11, 12	4.52E-03	1.05E-03
module5 (16)	interphase of mitotic cell cycle (< 0.001), DNA replication initiation (< 0.001)	3, 5, 7, 8	1, 2, 3, 5, 8, 9, 11, 12	1, 4, 5, 6, 7, 8, 9, 10, 11, 12	5, 7, 10, 11	5.28E-03	3.45E-04
module6 (38)	lipoprotein biosynthetic process and metabolic process (0.004), regulation of DNA metabolic process (0.005), chromosome organization (< 0.001)	1, 3, 5, 7	3, 4, 6, 7, 8, 9, 12	NA	6, 9	1.10E-03	2.88E-05
module7 (20)	nuclear division (< 0.001), cell-division (< 0.001), mitosis (< 0.001)	2, 3, 5, 8	3, 5, 7, 8, 9, 10, 12	1, 2, 3, 4, 5, 6, 8, 9, 10, 11	1, 2, 3, 4, 6, 7, 8, 10	2.10E-03	2.67E-04
module8 (9)	cell cycle (0.007), regulation of cell cycle (0.025)	3, 5, 7, 8	1, 2, 3, 4, 7, 8, 9, 11, 12	1, 3, 4, 5, 7, 8, 9, 10, 11	1, 2, 3, 4, 5, 6, 7, 8, 9, 11	1.01E-02	7.90E-04
module9 (35)	Glycosylation (< 0.001), mitotic cell cycle (< 0.001), nuclear division (< 0.001)	1, 5, 7, 8	1, 2, 4, 6, 8, 11	2	1, 2, 4, 5, 6, 7, 8, 11	5.20E-04	1.34E-05
module10 (14)	regulation of cell cycle (< 0.001), regulation of cell cycle process (0.001)	5, 7, 8, 12	1, 2, 3, 5, 7, 11, 12	3, 11	7, 11	6.55E-03	6.82 E-06
module11 (53)	cell cycle (0.043), nuclear migration along microtubule (0.012)	5, 7, 8, 10	1, 3, 4, 5, 8, 9, 10	NA	4, 5, 8, 9, 10	8.03E-05	8.70E-06
module12 (23)	mitotic recombination (< 0.001), DNA metabolic process (< 0.001)	1, 3, 5	2, 3, 4	10	1, 2, 3, 4, 5, 6, 7, 8, 10	2.27E-03	1.41E-04

As shown in Table 1, the function annotation analysis suggested that genes in the identified functional modules participate in broad biological process such as cell cycle, DNA replication or packaging, meiosis, regulation of transcription etc. For example, module 3 was highly enriched in DNA packaging; module 7 was enriched in cell-division cycle and mitosis; and DNA metabolic process was related to module 12. Although each functional module has multiple enriched annotations, but most annotations can be grouped into one or two clusters.

After grouping genes into functional modules, we applied step 2 to all functional modules, and obtained *X*_*i*_(*t*) and ${\hat{X}}_{i}^{\prime }\left(t\right),\;i=1,\cdots ,12$. Following the data augmentation strategy used in [13, 52, 53] and [54], we selected 300 time points from *X*_*i*_(*t*) and the first derivative ${\hat{X}}_{1}^{\prime }\left(t\right)$ for the module. Therefore, the sample size is *N* = 300. After substituting the estimates into single-index models, we built the full model for module 1, for instance, as follows.

$$\begin{array}{c} y_{1}=\eta_{1}(X{\left(t\right)}^{\text{T}}\beta_{0}^{\left[1\right]})+\varepsilon , \end{array}$$ (15)

where the response variable $y_{1}={\hat{X}}_{1}^{\prime }\left(t\right)$, the estimated first derivatives; **X**(*t*) = (*X*_1_(*t*), …, *X*_12_(*t*))^T^ are the population mean estimates of 12 functional modules; and $\beta_{0}^{\left[1\right]}={\left(\beta_{01}^{\left[1\right]},\ldots ,\beta_{012}^{\left[1\right]}\right)}^{\text{T}}$. Applying the PPrLS procedure given in step 3 to model (15), we detected significant variables **X**(*t*) and obtained nonzero ${\hat{\beta }}^{\left[1\right]}$. As a result, we identified the modules related to the gene-expression changes of the module 1. For a comparison, we also fitted *y* to **X**(*t*) by using a linear ODE model [13]

$$\begin{array}{c} y_{1}=X{\left(t\right)}^{\text{T}}\beta_{L0}^{\left[1\right]}+\varepsilon . \end{array}$$ (16)

We also selected X(t) and estimated $\beta_{L0}^{\left[1\right]}={\left(\beta_{L01}^{\left[1\right]},\ldots ,\beta_{L012}^{\left[1\right]}\right)}^{\text{T}}$ by applying the SCAD method to the linear ODE model (16).

**Fig 2:**
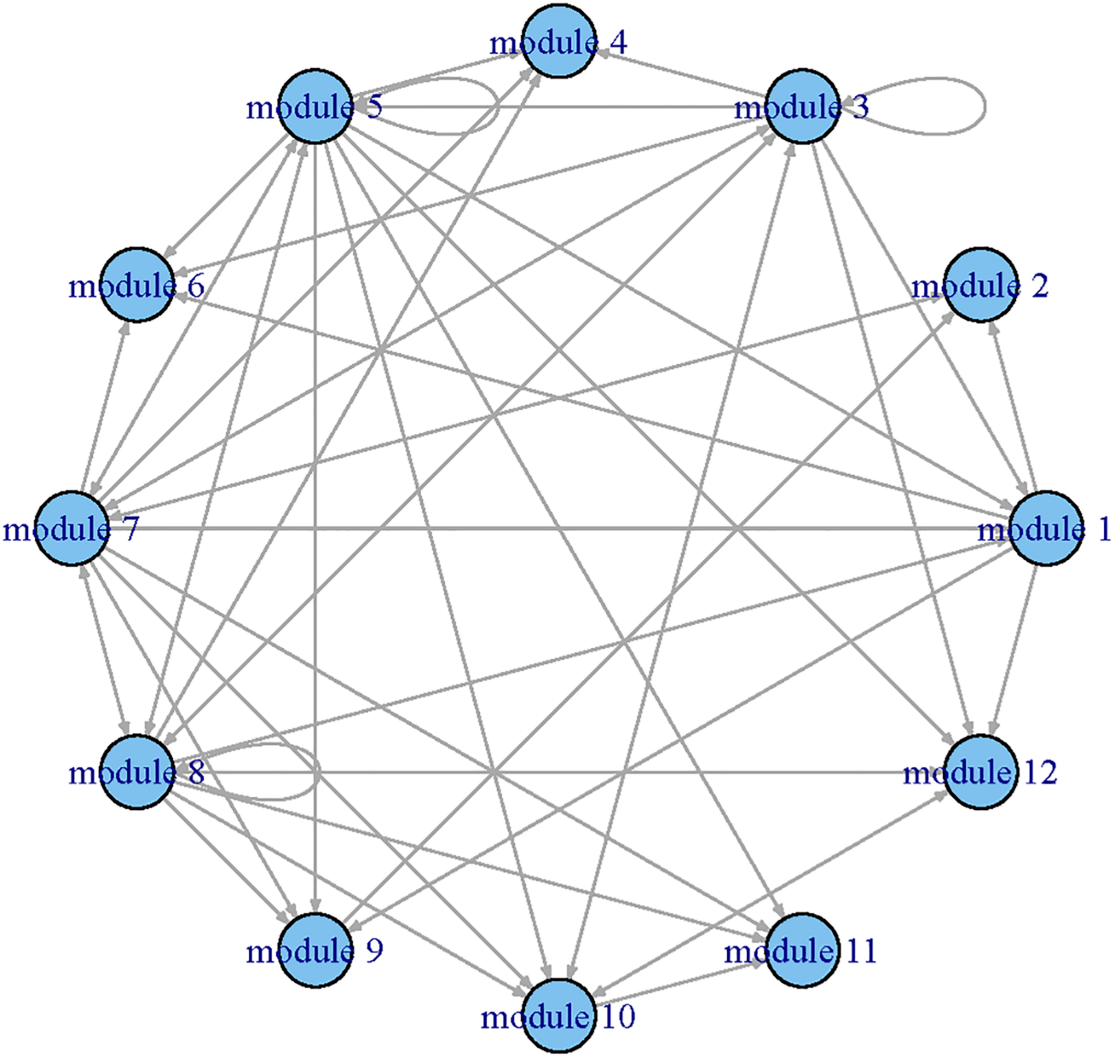
The GRN identified by the linear ODE models for the time course yeast cell data set. Each node represents a module and the arrows presents the direction of influence.

**Fig 3:**
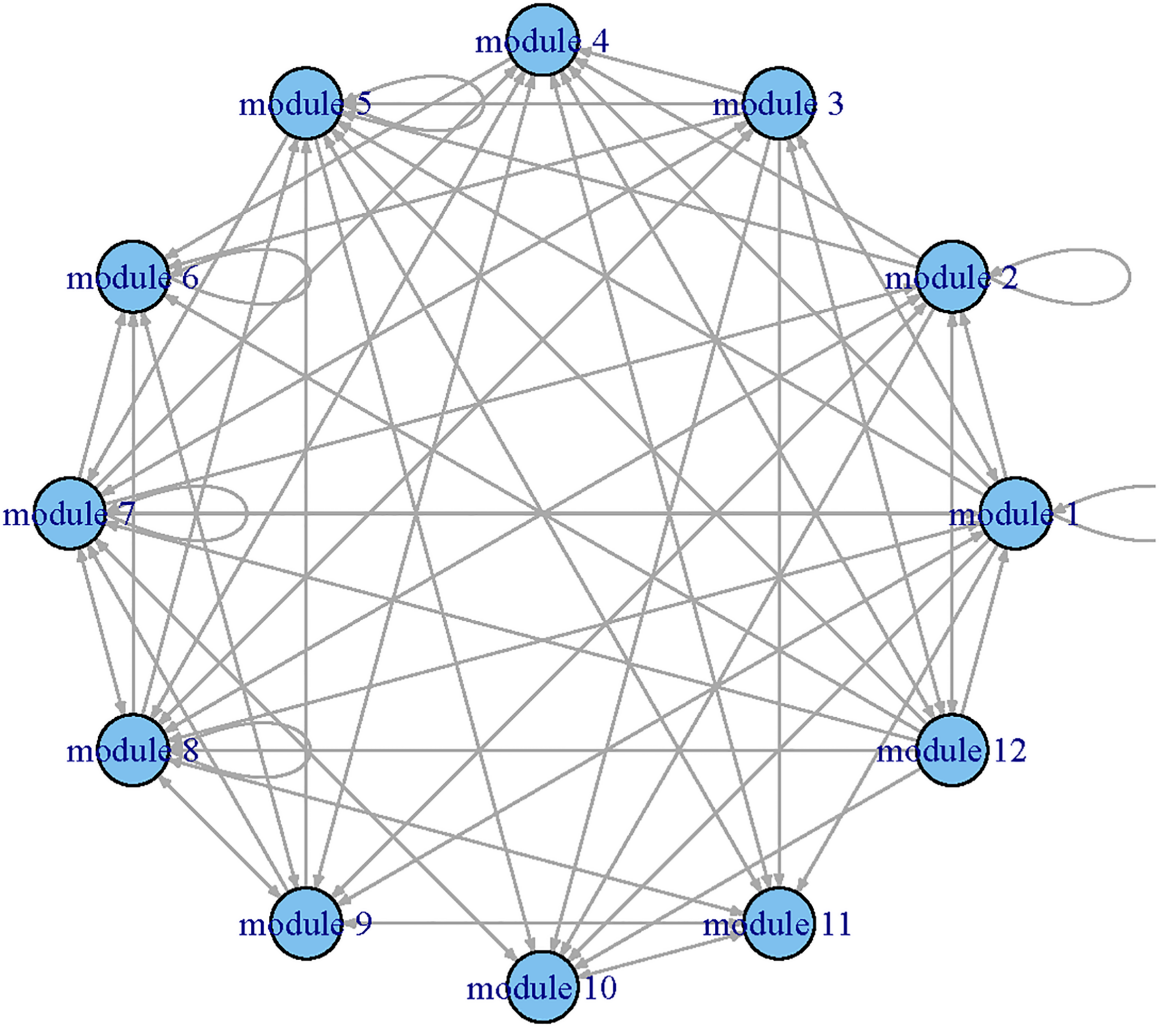
The GRN identified by the single-index ODE models for the time course yeast cell data set. Each node represents a module and the arrows presents the direction of influence.

Applying the procedure to all functional modules, we constructed a regulatory network among modules (Figs 2 and 3) and estimated their corresponding dynamic coefficients by both single-index and linear ODE models.

To compare the results provided by the single-index ODE and linear ODE models, we summarized the inward (significantly impact on) and outward (impacted by) regulatory relationships between modules in Table 1. The number of genes in each module was displayed in the parentheses. One can see that the residuals of sum squares (RSS) of single-index ODE models were smaller than those of the linear ODE models. We can also observe that the single-index ODE models selected more modules than the linear ODE models did. For example, the single-index ODE model indicated that module 2 was impacted by modules 1, 2, 7, 8, 9 and 12 of which only modules 1, 7 and 9 were selected by the linear ODE model. Both linear ODE and single-index ODE indicated that modules 3, 7 and 8 were important because they regulated more than 50% modules. We also noted that module 8 only included 9 genes. Further experiments are needed to explore these new discoveries in biological progression.

### A simulation study

In this part we conducted Monte Carlo simulation studies to validate the proposed procedure for the single-index ODE models. Due to the intensive computational cost, we designed a system with 7 ODEs, which include following linear and nonlinear forms. The simulation settings are data-driven because the gene expression pattern show sine and cosines patterns (Fig 1)

$$\begin{array}{ccc} & & \frac{dX_{1}\left(t\right)}{dt}=0.05*\left(\beta_{01}*X_{1}+\beta_{02}*X_{2}\right),\;\frac{dX_{2}\left(t\right)}{dt}=\text{cos}\left(\beta_{03}*X_{2}+\beta_{04}*X_{3}\right),\\ & & \frac{dX_{3}\left(t\right)}{dt}=\text{sin}\left(\beta_{05}*X_{2}+\beta_{06}*X_{3}\right),\;\frac{dX_{4}\left(t\right)}{dt}=0.1*\left(\beta_{07}*X_{2}+\beta_{08}*X_{4}\right),\\ & & \frac{dX_{5}\left(t\right)}{dt}=\text{sin}\left(\beta_{09}*X_{2}+\beta_{010}*X_{4}\right),\;\frac{dX_{6}\left(t\right)}{dt}=0.05*\text{exp}\left(\beta_{011}*X_{3}+\beta_{012}*X_{6}\right),\\ & & \frac{dX_{7}\left(t\right)}{dt}=0.2*\left(\beta_{013}*X_{2}+\beta_{014}*X_{3}\right),\;X_{p}\left(t_{0}\right)=X_{p0},\;p=1,\cdots ,7, \end{array}$$ (17)

where $\beta_{0}^{\left[1\right]}={\left(\beta_{01},\beta_{02},0,0,0,0,0\right)}^{\text{T}}={\left(0.707,0.707,0,0,0,0,0\right)}^{\text{T}}$, $\beta_{0}^{\left[2\right]}={\left(0,\beta_{03},\beta_{04},0,0,0,0\right)}^{\text{T}}={\left(0,0.555,-0.832,0,0,0,0\right)}^{\text{T}}$, $\beta_{0}^{\left[3\right]}={\left(0,\beta_{05},\beta_{06},0,0,0,0\right)}^{\text{T}}={\left(0,0.832,-0.555,0,0,0,0\right)}^{\text{T}}$, $\beta_{0}^{\left[4\right]}={\left(0,\beta_{07},0,\beta_{08},0,0,0\right)}^{\text{T}}={\left(0,0.600,0,-0.800,0,0,0\right)}^{\text{T}}$, $\beta_{0}^{\left[5\right]}={\left(0,\beta_{09},0,\beta_{010},0,0,0\right)}^{\text{T}}={\left(0,0.894,0,0.447,0,0,0\right)}^{\text{T}}$, $\beta_{0}^{\left[6\right]}={\left(0,0,\beta_{011},0,0,\beta_{012},0\right)}^{\text{T}}={\left(0,0,0.894,0,0,-0.447,0\right)}^{\text{T}}$, and $\beta_{0}^{\left[7\right]}={\left(0,\beta_{013},\beta_{014},0,0,0,0\right)}^{\text{T}}={\left(0,0.894,-0.447,0,0,0,0\right)}^{\text{T}}$.

**Fig 4:**
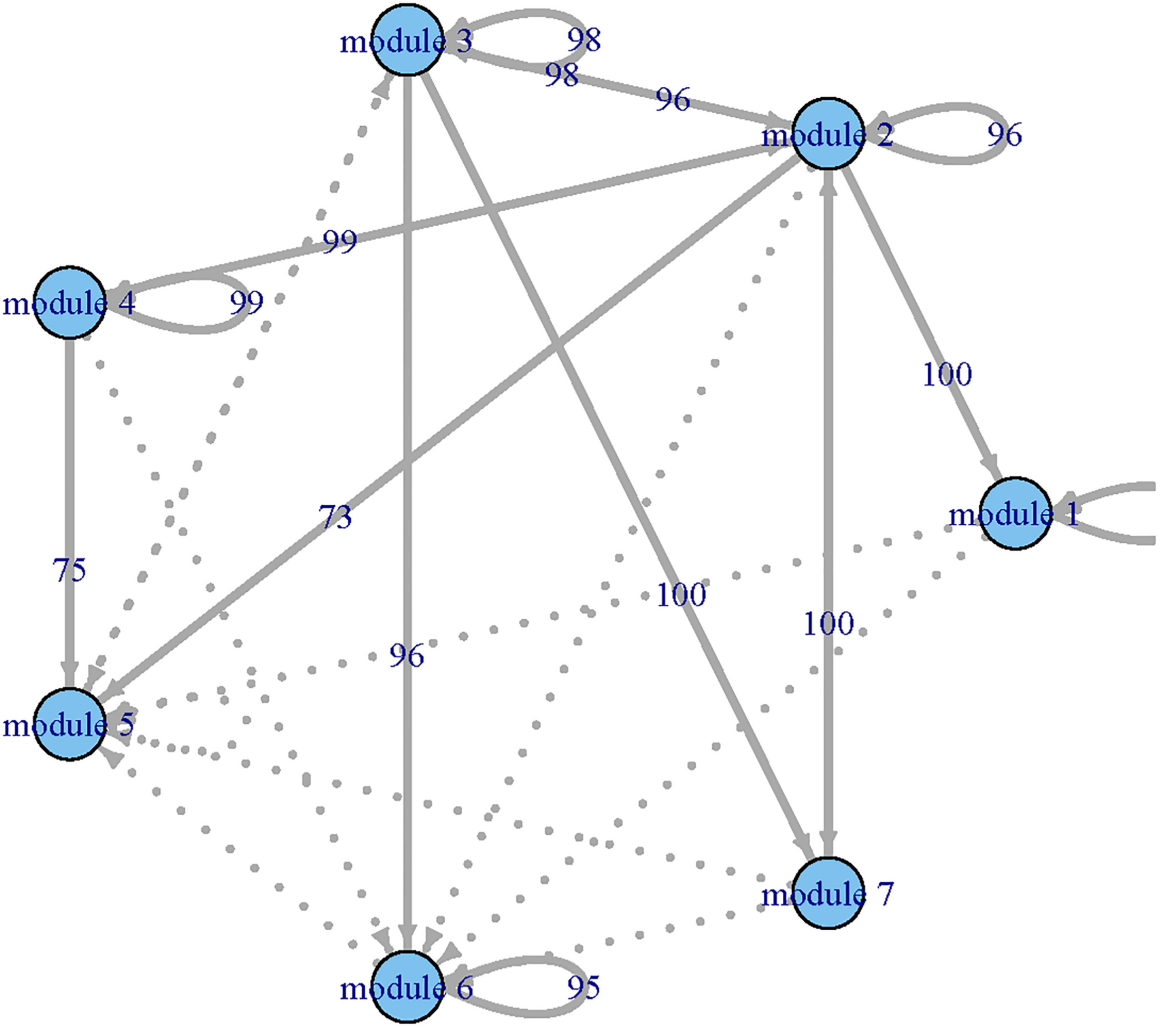
The constructed gene regulatory networks for simulation studies with *N* = 180 and 100 iterations. Solid lines: the true connections, numbers present: the times correctly identified using our procedure in 100 iteration, dots line: incorrectly identified connections.

**Fig 5:**
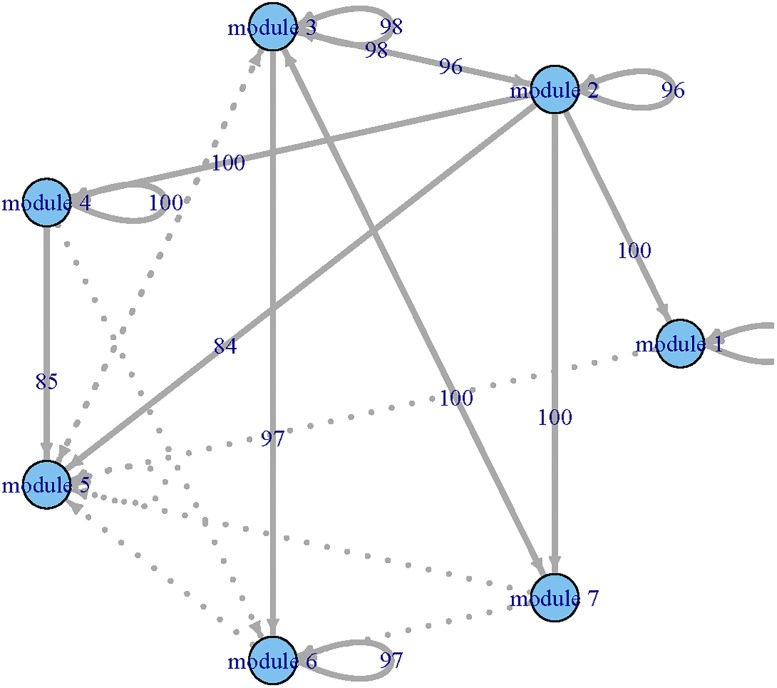
The constructed gene regulatory networks for simulation studies with *N* = 288 and 100 iterations. The legend is the same as in Fig 4.

**Fig 6:**
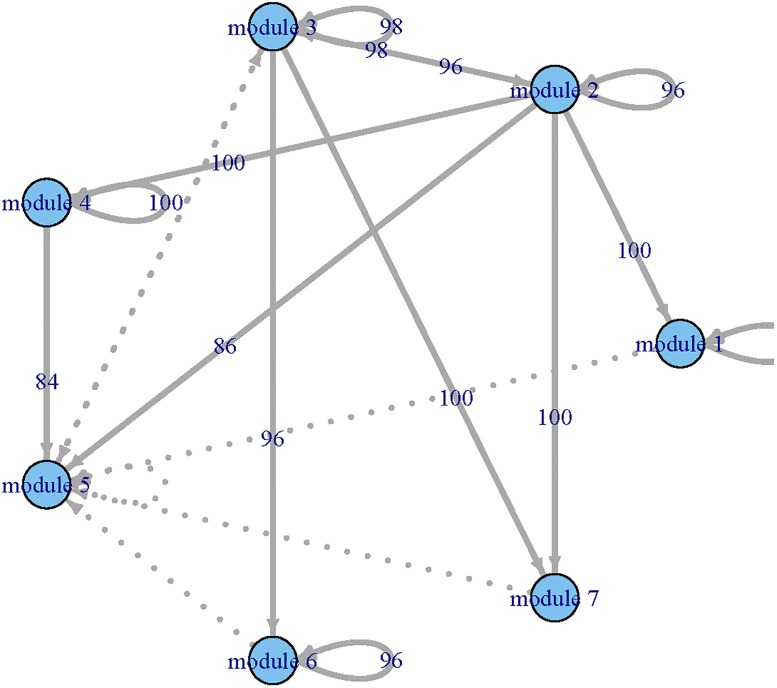
The constructed gene regulatory networks for simulation studies with *N* = 360 and 100 iterations. The legend is the same as in Fig 4.

Given initial values *X*_*p*0_, *p* = 1, …, 7, we can numerically solve the above ODE system and obtain the numerical solution *X*_*p*_(*t*), *p* = 1, …, 7. In this simulation study, we first generated initial values *X*_*p*_(0), *p* = 1, …, 7 by using

$$\begin{array}{c} X_{p0}=X_{0}+0.5*e_{p},\;p=1,\ldots ,7, \end{array}$$

where *e*_*p*_ follows *N*(0, 1) and *X*_0_ = (0.7628, 0.6789, 1.2351, 0.6170, 2.7800, 0.2906, 0.4441). We then numerically solved the ODE system (17) and output *X*_*p*_(*t*), *p* = 1, …, 7, using three different schedules: equally spaced time points on the ranges of [0, 18], but three different intervals between time points. As a result, we simulated seven population means *X*_*p*_(*t*_*i*_), *p* = 1, …, 7, *i* = 1, …, *N* with sample sizes *N* = 180, 288, 360. After generating the population mean curves, we used the spline method to estimate the first derivatives, which are denoted by ${\hat{X}}_{p}^{\prime }\left(t\right)$, *p* = 1, …, 7. For notation simplicity, we gave the model structure and estimation procedure for the first ODE (*k* = 1). The same procedure can be applied to the rest of the ODEs. Substituting the generated *X*_*p*_(*t*), *p* = 1, …, 7 and estimated ${\hat{X}}_{1}^{\prime }\left(t\right)$ into the single-index ODE models, we obtained the largest model for the first ODE as follows:

$$\begin{array}{c} {\hat{X}}_{1}^{\prime }\left(t_{i}\right)=\eta_{1}(X{\left(t_{i}\right)}^{\text{T}}\beta_{0}^{\left[1\right]})+\varepsilon_{i},\;i=1,\ldots ,N, \end{array}$$

where $\beta_{0}^{\left[1\right]}={\left(\beta_{01}^{\left[1\right]},\ldots ,\beta_{07}^{\left[1\right]}\right)}^{\text{T}}$ and **X**(*t*_*i*_) = (*X*_1_(*t_i_*), ⋯, *X*_7_(*t_i_*))^T^, *i* = 1, ⋯, *N*. Applying the procedure given in Step 3, we selected **X**(*t*) and estimated $\beta_{0}^{\left[1\right]}$ for the first ODE. Applying the same procedure to the other six ODEs, we estimated $\beta_{0}^{\left[k\right]}$, *k* = 2, …, 7. As a result, we constructed GRN for the simulated functional modules. We repeated the same procedure 100 time and summarized the ${\text{MSE}}_{q}=\sum_{j=1}^{100}{\left({\hat{\beta }}_{qj}-\beta_{0q}\right)}^{2}/100$ and ${\text{ARE}}_{q}=\sum_{\text{j}=1}^{100}\frac{|{\hat{\beta }}_{qj}-\beta_{0q}|}{|\beta_{0q}|},\;q=1,\ldots ,14$, where ${\hat{\beta }}_{qj}$ is the estimated *β*_*q*_ for *j*_*th*_ iteration. In Table 2, “overfitted (O)” represents extra variables; “underfitted (U)” represents incorrectly deleting necessary variables. We can see that the PPrLS method can correctly select the variables for most cases in terms of the number of correctly fitted model. Larger sample sizes lead to better performance. For ODEs with a linear form, namely ODE1, ODE4, and ODE7, both variable selection and parameter estimation procedures have good performance when the sample size is 180. For the nonlinear case, with the increase of the sample size, both variable selection and parameter estimation tend to work better. In addition, we reported the 10% trimmed MSE and ARE (discarding 5% of the lowest and the highest values). Meanwhile, we constructed networks among simulated functional modules for each iteration (see Figs 4, 5 and 6). The thick lines represents true connection, and the numbers present the times which were found by our method in 100 iterations. From Figs 4, 5 and 6, we can see that the constructed GRN match the true network in most cases.

**Table 2:** The simulation results for the SCAD method for scenarios with different sample sizes based on 100 replications. The simulation results for the SCAD method for scenarios with different sample sizes based on 100 replications. Correctly fitted (C); underfitted (U); overfitted(O).

ODE	$\hat{\beta }’\text{s}$	C	U	O	MSE	MSE_*trim*_	ARE(%)	ARE_*trim*_(%)
	*N* = 180							
1	${\hat{\beta }}_{1}$	100	0	0	< 0.001	< 0.001	0.005	0.004
	${\hat{\beta }}_{2}$				< 0.001	< 0.001	0.005	0.004
2	${\hat{\beta }}_{3}$	96	0	3	0.012	< 0.001	4.012	0.013
	${\hat{\beta }}_{4}$				0.028	< 0.001	4.005	0.006
3	${\hat{\beta }}_{5}$	97	1	2	0.014	< 0.001	2.271	0.006
	${\hat{\beta }}_{6}$				0.007	< 0.001	2.366	0.013
4	${\hat{\beta }}_{7}$	99	0	1	0.004	< 0.001	1.025	0.024
	${\hat{\beta }}_{8}$				0.006	< 0.001	1.014	0.013
5	${\hat{\beta }}_{9}$	65	6	24	0.295	0.255	35.181	32.363
	${\hat{\beta }}_{10}$				0.064	0.057	32.669	30.219
6	${\hat{\beta }}_{11}$	93	2	3	0.053	0.005	6.443	1.105
	${\hat{\beta }}_{12}$				0.014	0.003	6.811	1.538
7	${\hat{\beta }}_{13}$	100	0	0	< 0.001	< 0.001	0.004	0.003
	${\hat{\beta }}_{14}$				< 0.001	< 0.001	0.016	0.014
	*N* = 288							
1	${\hat{\beta }}_{1}$	100	0	0	< 0.001	< 0.001	0.002	0.001
	${\hat{\beta }}_{2}$				< 0.001	< 0.001	0.002	0.001
2	${\hat{\beta }}_{3}$	96	0	4	0.012	< 0.001	4.003	0.003
	${\hat{\beta }}_{4}$				0.028	< 0.001	4.001	0.001
3	${\hat{\beta }}_{5}$	97	1	2	0.014	< 0.001	2.264	0.002
	${\hat{\beta }}_{6}$				0.007	< 0.001	2.49	0.003
4	${\hat{\beta }}_{7}$	100	0	0	< 0.001	< 0.001	0.007	0.007
	${\hat{\beta }}_{8}$				< 0.001	< 0.001	0.004	0.004
5	${\hat{\beta }}_{9}$	77	5	13	0.166	0.14	21.242	18.046
	${\hat{\beta }}_{10}$				0.055	0.035	23.601	18.503
6	${\hat{\beta }}_{11}$	96	1	2	0.024	< 0.001	3.167	0.001
	${\hat{\beta }}_{12}$				0.006	< 0.001	3.19	0.003
7	${\hat{\beta }}_{13}$	100	0	0	< 0.001	< 0.001	0.001	0.001
	${\hat{\beta }}_{14}$				< 0.001	< 0.001	0.004	0.004
	*N* = 360							
1	${\hat{\beta }}_{1}$	100	0	0	< 0.001	< 0.001	0.001	0.001
	${\hat{\beta }}_{2}$				< 0.001	< 0.001	0.001	0.001
2	${\hat{\beta }}_{3}$	96	0	4	0.012	< 0.001	4.002	0.002
	${\hat{\beta }}_{4}$				0.028	< 0.001	4.001	0.001
3	${\hat{\beta }}_{5}$	97	1	2	0.014	< 0.001	2.278	0.001
	${\hat{\beta }}_{6}$				0.007	< 0.001	2.402	0.002
4	${\hat{\beta }}_{7}$	100	0	0	< 0.001	< 0.001	0.005	0.005
	${\hat{\beta }}_{8}$				< 0.001	< 0.001	0.003	0.003
5	${\hat{\beta }}_{9}$	78	5	10	0.15	0.122	19.267	15.852
	${\hat{\beta }}_{10}$				0.039	0.032	20.493	17.214
6	${\hat{\beta }}_{11}$	96	0	4	0.032	< 0.001	4	< 0.001
	${\hat{\beta }}_{12}$				0.008	< 0.001	4.002	0.002
7	${\hat{\beta }}_{13}$	100	0	0	< 0.001	< 0.001	0.001	< 0.001
	${\hat{\beta }}_{14}$				< 0.001	< 0.001	0.002	0.002

## Conclusions and discussions

In this paper, we have proposed single-index ODE models and developed a procedure to select variables and estimate parameters. The procedure has further been used to analyze a time-course data set with the aim of exploring the module based and regulator-regulator interactions. We found the interactions identified by using single-index ODE were more accurate, i.e., the linear ODE models overlooked some confirmed regulator-regulator interactions [55]. We took module 12 as an example. MBP1 is a DNA-binding protein that forms MBF complex; a protein complex that binds to the Mlu1 cell cycle box promoter element. [56, 57] showed that MBP1 is topologically related to transcription factors, including SWI4 in *Saccharomyces cerevisiae*. In addition, there is physical and genetic evidence that MBP1 interacts with SKN7, a transcription factor [58]. These two interactions are identified as potential interactions in module 12 by single-index ODE models, but are overlooked by the linear ODE models.

The advantages of our method include (i) single-index ODE models can fit the data better than linear ODE models; (ii) the interactions found by single-index ODE models can cover most of the interactions identified by linear ODE models for some of the modules; and (iii) our method is computationally efficient because we can select significant modules and estimate index coefficients simultaneously.

Similar to the linear ODE model, our method needs estimated population means and their corresponding first derivatives, which may be treated as the limitation of the proposed procedure. The PPrLS estimator has good performance in identifying significant modules. But new stable techniques are still needed to group genes to reduce the gene cluster uncertainty because cluster assignment still plays an important role in enhancing the usefulness of this research. It is worth noting that the PPrLS estimates may not be most efficient in terms of estimation accuracy because PPrLS estimation is a nonparametric method and inherits error if the data contain a large noise. Regulator-regulator interaction exploration depends on the knowledge of gene-regulator relationship, which we study. So the proposed method may provide valuable insights into complicated biological processes with understanding gene-gene and gene-regulator relationships. Overall, our procedure is useful to single out high level (module based) and potential regulator-regulator interactions which are helpful to provide guidance for tedious and costly experiments.

## Supporting information

S1 Table All enriched functional annotations. (PDF)

S2 Table The estimated regression coefficients for every functional modules using single-index and linear ODE models. (PDF)

S1 File Large-sample properties of the PPrLS procedure and discussion of computation cost. (PDF)

S2 File Clustered data. (TXT)

S3 File Estimated coefficients using the proposed model and methods. (TXT)

S4 File Estimated coefficients using the linear ODE model. (TXT)

S5 File Functional annotations. (XLS)

S6 File R code for clustering. (R)

S7 File R code for drawing Fig 3. (R)
