# Supplementary material for: Using single-index ODEs to study dynamic gene regulatory network
Source: PLoS One. 2018 Feb 23;13(2):e0192833. doi: 10.1371/journal.pone.0192833 (PMC5825071; doi:10.1371/journal.pone.0192833)
Supplement: S1 Table — (PDF) [file pone.0192833.s001.pdf]

S1 Table. The regulator-regulator interactions identified by single-index and linear ODE models. Regulators: regulators related genes included in a certain module; Single-index ODE: regulators identified by single-index ODE models only; Both: regulators found by both linear and single-index ODE models, Linear ODE: regulators identified by linear ODE models only.

Module	Model	Regulators
Module1	Regulators	NDT80,MIG1,MOT3,RGT1,MCM1,SWI6
	Single-index ODE	NDT80,MIG1,MOT3,RGT1,SKN7
	both	MCM1,SWI6,HIR1,HIR2,HIR3,TEC1,SWI4,FKH1,FKH2<br>XBP1,PDR1,PDR3,RTG1,HAA1,RLM1,STE12,RME1,MBP1
	Linear ODE	MSN4,THI2,SOK2,SWI5,ROX1,SPT23
Module2	Regulators	MSN2,MSN4,GCN4,PHO2
	Single-index ODE	MSN2,MSN4,GCN4,PHO2,STE12,RME1,MBP1,SKN7
	both	NDT80,MIG1,MOT3,RGT1,MCM1,SWI6,FKH1,FKH2<br>NDD1,XBP1,PDR1,PDR3,RTG1,HAA1,RLM1,SWI4,TEC1
	Linear ODE	
Module3	Regulators	HIR1,HIR2,HIR3,TEC1,SWI4
	Single-index ODE	NDT80,MIG1,MOT3,RGT1,SKN7
	both	MCM1,SWI6,FKH1,FKH2,NDD1,XBP1,PDR1<br>PDR3,RTG1,HAA1,RLM1,SWI4,STE12,TEC1,RME1,MBP1
	Linear ODE	HIR1,HIR2,HIR3,UME6,ZAP1
Module4	Regulators	MSN2,MSN4,RFX1,MBP1,SWI6,REB1,ZAP1,RLM1,UME6
	Single-index ODE	NDT80,MIG1,MOT3,RGT1,MSN2,GCN4,PHO2,ABF1,SKN7
	both	MCM1,SWI6,MSN4,HIR1,HIR2,HIR3,TEC1,SWI4, FKH1,FKH2<br>NDD1,XBP1,PDR1,PDR3,RTG1 HAA1,RLM1,STE12,RME1,MBP1
	Linear ODE	THI2,SOK2,SWI5,ROX1,SPT23
Module5	Regulators	MCM1,RLM1,MSN4,STE12,PDR1,THI2,SOK2,SWI5,ROX1,SPT23
	Single-index ODE	NDT80,MIG1,MOT3,RGT1,MSN2,GCN4,PHO2,ABF1,SKN7
	both	MCM1,SWI6,MSN4,HIR1,HIR2,HIR3,TEC1,SWI4<br>RLM1,STE12,PDR1,THI2,SOK2,SWI5,ROX1,SPT23,XBP1,RME1,MBP1
	Linear ODE	FKH1,FKH2,NDD1,PDR3,RTG1,HAA1
Module6	Regulators	PDR1,ZAP1
	Single-index ODE	MSN2,RFX1,MBP1,REB1,ZAP1,UME6,RME1,SKN7
	both	HIR1,HIR2,HIR3,TEC1,SWI4,MSN4,SWI6,RLM1,PDR1<br>FKH1,FKH2,MCM1,NDD1,XBP1,PDR3,RTG1,HAA1,STE12
	Linear ODE	NDT80,MIG1,MOT3,RGT1,THI2,SOK2,SWI5,ROX1,SPT23
Module7	Regulators	FKH1,FKH2,MCM1,NDD1,XBP1,PDR1,PDR3,RTG1,HAA1,RLM1
	Single-index ODE	FKH1,FKH2,NDD1,PDR3,RTG1,HAA1,UME6,ZAP1,SKN7
	both	HIR1,HIR2,HIR3,TEC1,SWI4,MCM1,RLM1,MSN4,STE12<br>PDR1,THI2,SOK2,SWI5,ROX1,SPT23,XBP1,SWI6,RME1,MBP1
	Linear ODE	MSN2,GCN4,PHO2
Module8	Regulators	SWI4,STE12,SWI6,TEC1,XBP1,RME1,MBP1
	Single-index ODE	NDT80,MIG1,MOT3,RGT1,MSN2,GCN4,PHO2,RFX1,REB1,ZAP1,UME6,ABF1,SKN7
	both	MCM1,SWI6,MSN4,HIR1,HIR2,HIR3,TEC1,SWI4,MBP1<br>RLM1,FKH1,FKH2,NDD1,XBP1,PDR1,PDR3,RTG1,HAA1,STE12,RME1
	Linear ODE	THI2,SOK2,SWI5,ROX1,SPT23
Module9	Regulators	TEC1,RLM1,SWI4
	Single-index ODE	MSN2,GCN4,PHO2,RFX1,REB1,ZAP1,UME6,ABF1

	both	NDT80,MIG1,MOT3,RGT1,MCM1,SWI6,MSN4,MBP1<br>RLM1,PDR1,SWI4,STE12,TEC1,XBP1,RME1
	Linear ODE	THI2,SOK2,SWI5,ROX1,SPT23,FKH1,FKH2,NDD1,PDR3,RTG1,HAA1
Module10	Regulators	TEC1,UME6,HAA1,MCM1,XBP1,ZAP1
	Single-index ODE	NDT80,MIG1,MOT3,RGT1,MSN2,GCN4,PHO2,HIR1,HIR2,HIR3,ABF1
	both	MCM1,SWI6,MSN4,TEC1,SWI4,RLM1,STE12,PDR1,THI2<br>SOK2,SWI5,ROX1,SPT23,FKH1,FKH2,NDD1,XBP1,PDR3,RTG1,HAA1,SKN7
	Linear ODE	RME1,MBP1
Module11	Regulators	ABF1
	Single-index ODE	NDT80,MIG1,MOT3,RGT1,HIR1,HIR2,HIR3,MSN2,RFX1,REB1
	both	MCM1,SWI6,TEC1,SWI4,MSN4,MBP1,ZAP1,RLM1,UME6<br>STE12,PDR1,THI2,SOK2,SWI5,ROX1,SPT23,XBP1,RME1,HAA1
	Linear ODE	FKH1,FKH2,NDD1,PDR3,RTG1
Module12	Regulators	SKN7,SWI4
	Single-index ODE	MSN2,GCN4,PHO2,RFX1,MBP1,REB1
	both	MSN4,HIR1,HIR2,HIR3,TEC1,SWI4,SWI6,ZAP1,RLM1,UME6
	Linear ODE	NDT80,MIG1,MOT3,RGT1,MCM1,STE12,PDR1<br>THI2,SOK2,SWI5,ROX1,SPT23,HAA1,XBP1
