# Supplementary material for: Using single-index ODEs to study dynamic gene regulatory network
Source: PLoS One. 2018 Feb 23;13(2):e0192833. doi: 10.1371/journal.pone.0192833 (PMC5825071; doi:10.1371/journal.pone.0192833)
Supplement: S2 Table — (PDF) [file pone.0192833.s002.pdf]

Module	Model	$\widehat{\boldsymbol{\beta}}$	1	2	3	4	5	6	7	8	9	10	11	12	RSS
module1	Linear ODE	$\widehat{\boldsymbol{\beta}}_L^{[1]}$	0.00	0.00	-0.18	0.00	0.24	0.00	-0.31	-0.49	0.00	0.00	0.00	0.00	3.07E-03
	Single-index ODE	$\widehat{\boldsymbol{\beta}}^{[1]}$	0.14	0.00	-0.28	0.00	0.00	0.00	0.23	0.34	-0.41	0.00	0.00	-0.76	1.41E-04
module2	Linear ODE	$\widehat{\boldsymbol{\beta}}_L^{[2]}$	-0.16	0.00	0.00	0.00	0.00	0.00	0.07	0.00	-0.25	0.00	0.00	0.00	5.20E-04
	Single-index ODE	$\boldsymbol{\beta}^{[2]}$	0.73	-0.37	0.00	0.00	0.00	0.00	-0.14	-0.22	0.40	0.00	0.00	-0.33	3.38E-05
module3	Linear ODE	$\widehat{\boldsymbol{\beta}}_L^{[3]}$	0.00	0.00	-0.18	0.00	0.00	0.00	-0.23	0.55	0.00	0.08	0.00	0.00	3.37E-03
	Single-index ODE	$\widehat{\boldsymbol{\beta}}^{[3]}$	0.73	0.00	0.00	0.00	0.00	0.00	-0.49	0.48	0.00	0.00	0.00	0.01	3.58E-03
module4	Linear ODE	$\widehat{\boldsymbol{\beta}}_L^{[4]}$	0.00	0.00	-0.22	0.00	0.28	0.00	-0.11	-0.04	0.00	0.00	0.00	0.00	4.52E-03
	Single-index ODE	$\widehat{\boldsymbol{\beta}}^{[4]}$	0.14	-0.53	0.11	0.00	0.00	0.00	-0.30	-0.31	-0.34	0.00	0.47	0.42	1.05E-03
module5	Linear ODE	$\widehat{\boldsymbol{\beta}}_L^{[5]}$	0.00	0.00	-0.26	0.00	-0.18	0.00	0.48	-0.18	0.00	0.00	0.00	0.00	5.28E-03
	Single-index ODE	$\widehat{\boldsymbol{\beta}}^{[5]}$	0.09	-0.12	-0.37	0.00	0.11	0.00	0.00	0.37	0.38	0.00	0.42	-0.61	3.45E-04
module6	Linear ODE	$\widehat{\boldsymbol{\beta}}_L^{[6]}$	-0.09	0.00	-0.19	0.00	0.05	0.00	-0.06	0.00	0.00	0.00	0.00	0.00	1.10E-03
	Single-index ODE	$\widehat{\boldsymbol{\beta}}^{[6]}$	0.00	0.00	0.28	0.19	0.00	-0.25	-0.26	-0.49	-0.35	0.00	0.00	0.63	2.88E-05
module7	Linear ODE	$\widehat{\boldsymbol{\beta}}_L^{[7]}$	0.00	-0.22	-0.10	0.00	-0.53	0.00	0.00	0.17	0.00	0.00	0.00	0.00	2.10E-03
	Single-index ODE	$\widehat{\boldsymbol{\beta}}^{[7]}$	0.00	0.00	0.39	0.00	-0.20	0.00	-0.13	-0.38	-0.33	-0.31	0.00	0.67	2.67E-04
module8	Linear ODE	$\widehat{\boldsymbol{\beta}}_L^{[8]}$	0.00	0.00	-0.26	0.00	0.52	0.00	-0.51	-0.34	0.00	0.00	0.00	0.00	1.01E-02
	Single-index ODE	$\widehat{\boldsymbol{\beta}}^{[8]}$	0.20	-0.58	0.11	0.15	0.00	0.00	-0.25	-0.39	-0.42	0.00	0.32	0.30	7.90E-04
module9	Linear ODE	$\widehat{\boldsymbol{\beta}}_L^{[9]}$	0.12	0.00	0.00	0.00	0.03	0.00	-0.16	0.08	0.00	0.00	0.00	0.00	5.20E-04
	Single-index ODE	$\widehat{\boldsymbol{\beta}}^{[9]}$	0.27	-0.74	0.00	0.28	0.00	0.23	0.00	-0.32	0.00	0.00	-0.37	0.00	1.34E-05
module10	Linear ODE	$\widehat{\boldsymbol{\beta}}_L^{[10]}$	0.00	0.00	0.00	0.00	-0.33	0.00	0.26	-0.07	0.00	0.00	0.00	0.43	6.55E-03
	Single-index ODE	$\widehat{\boldsymbol{\beta}}^{[10]}$	0.34	-0.35	-0.13	0.00	0.17	0.00	-0.24	0.00	0.00	0.00	0.67	-0.46	6.82E-06
module11	Linear ODE	$\widehat{\boldsymbol{\beta}}_L^{[11]}$	0.00	0.00	0.00	0.00	-0.07	0.00	0.05	0.14	0.00	-0.20	0.00	0.00	8.03E-05
	Single-index ODE	$\widehat{\boldsymbol{\beta}}^{[11]}$	0.14	0.00	-0.19	-0.24	-0.15	0.00	0.00	0.31	0.81	-0.33	0.00	0.00	8.70E-06
module12	Linear ODE	$\widehat{\boldsymbol{\beta}}_L^{[12]}$	-0.37	0.00	-0.41	0.00	0.06	0.00	0.00	0.00	0.00	0.00	0.00	0.00	2.27E-03
	Single-index ODE	$\widehat{\boldsymbol{\beta}}^{[12]}$	0.00	0.83	0.49	0.26	0.00	0.00	0.00	0.00	0.00	0.00	0.00	0.00	1.41E-04

S2 Table. The estimated regression coefficients for every functional modules using single-index and linear ODE models. $\widehat{\boldsymbol{\beta}}_L^{[k]},\ k = 1, \ldots, 12$ are the estimated coefficients using linear ODE models; $\widehat{\boldsymbol{\beta}}^{[k]},\ k = 1, \ldots, 12$ are the estimated coefficients using single-index ODE models.
