# Supplementary material for: Using single-index ODEs to study dynamic gene regulatory network
Source: PLoS One. 2018 Feb 23;13(2):e0192833. doi: 10.1371/journal.pone.0192833 (PMC5825071; doi:10.1371/journal.pone.0192833)
Supplement: S1 File — (PDF) [file pone.0192833.s003.pdf]

S1 File. Large-sample properties of the PPrLS procedure

We now investigate the large-sample properties of the PPrLS procedure and give the proof, which enables us to use the procedure for analysis of the gene expression data.

For sparse models, we consider a situation in which most of the regression coefficients are exactly zero. Without loss of generality, for the k-th single-index link function $\eta_k(\cdot)$, $k = 1, \ldots, p$, we assume that the first q_k functional modules $X_l(t)$, $l = 1, \ldots, q_k$, $k = 1, \ldots, p$ are relevant to $\eta_k(\cdot)$, and the remaining $p - q_k$ modules $X_s(t)$, $s = q_k + 1, \ldots, p$ are not related to $\eta_k(\cdot)$. Let $\boldsymbol{\beta}_0^{[k]} = \left(\boldsymbol{\beta}_{01}^{[k]\mathrm{T}}, \boldsymbol{\beta}_{02}^{[k]\mathrm{T}}\right)^{\mathrm{T}}$, where $\boldsymbol{\beta}_{01}^{[k]}$ consists of the q_k nonzero components of $\boldsymbol{\beta}_0^{[k]}$ and $\boldsymbol{\beta}_{02}^{[k]}$ is a $(p - q_k) \times 1$-zero vector. Moreover, let $X_{[q_k]}(t) = (X_1(t), \ldots, X_{q_k}(t))^{\mathrm{T}}$. In the following, we assume the following conditions and explore the consistency and asymptotic normality for PPrLS estimators.

Assumption A:

A1 The third derivatives $X_j^{(3)}(t)$s are continuous on $[0, 1]$.

A2 The kernel function $K(\cdot)$ is symmetric about zero and is supported on $[-1, 1]$.

A3 The bandwidth $h = h_N = N^{-1/7} a_N$ is a sequence satisfying $h \to 0$ as $N \to \infty$, where a_N is a sequence tending to 0 more slowly than $\log^{-1} N$.

A4 t_is are $i.i.d$ and have a common compact support and their density function, $f(t)$, is bounded away from zero and has bounded and continuous second derivatives.

A5 The density function $f_{\boldsymbol{\beta}^{[k]}}(u)$ of $X(t)^{\mathrm{T}}\boldsymbol{\beta}^{[k]}$ and its derivatives up to the third order are bounded on $\mathbb{R}$ for all $\boldsymbol{\beta} \in \mathfrak{H}_N = \left\{\boldsymbol{\beta} : \|\boldsymbol{\beta}^{[k]} - \boldsymbol{\beta}_0^{[k]}\| \leq C_1 N^{-1/2+c_0}\right\}$ for $c_0 < 1/20$ and some positive constant C_1. Moreover, $E|X_k(t_i)|^3 < \infty$, and $E|X_k'(t_i)|^3 < \infty$ for $k = 1, \ldots, p$, $i = 1, \ldots, N$.

A6 The functions $\eta_k(X(t)^{\mathrm{T}}\boldsymbol{\beta}^{[k]})(k = 1, \ldots, p)$ and their derivatives up to third order are bounded on $\mathbb{R}$ for all $\boldsymbol{\beta}^{[k]} \in \mathfrak{H}_N$.

A7 The regularity parameters $\lambda_j^{[k]}$s satisfy $\lambda_j^{[k]} \to 0$ and $N^{-1/2}/\lambda_j^{[k]} \to 0$; moreover,

$$\lim \inf_{N\to\infty} \lim \inf_{\beta_j^{[k]}\to 0^+} p'_{\lambda_j^{[k]}}(|\beta_j^{[k]}|)/\lambda_j^{[k]} > 0.$$

The foregoing regularity Conditions A1-A4 were used by [1]. Conditions A5-A6 are standard assumptions in nonparametric kernel smoothing estimation. Condition A7 is a usual condition for a SCAD-variable selection procedure, see [2].

Theorem 1 *Assuming that Conditions A1-A7 hold, we have*

- *With probability tending to 1, the penalized estimator* $\widehat{\boldsymbol{\beta}}_{02}^{[k]} = \mathbf{0}_{p-q_k}$*;*
- *The nonzero penalized estimator* $\widehat{\boldsymbol{\beta}}_{01}^{[k]}$ *is asymptotically normal:*

$$\sqrt{N}\left[\left(\widehat{\boldsymbol{\beta}}_{01}^{[k]} - \boldsymbol{\beta}_{01}^{[k]}\right) + \left(\mathcal{B}_{[k]} + \Sigma_{\lambda^{[k]}}\left(\boldsymbol{\beta}_{01}^{[k]}\right)\right)^{-1}\mathcal{R}_{\lambda^{[k]}}\left(\boldsymbol{\beta}_{01}^{[k]}\right)\right]$$

$$\xrightarrow{L} N\left(\mathbf{0}_{q_k\times 1}, \sigma^2\left(\mathcal{B}_{[k]} + \Sigma_{\lambda^{[k]}}\left(\boldsymbol{\beta}_{01}^{[k]}\right)\right)^{-1}\mathcal{B}_{[k]}\left(\mathcal{B}_{[k]} + \Sigma_{\lambda^{[k]}}\left(\boldsymbol{\beta}_{01}^{[k]}\right)\right)^{-1}\right),$$

where

$$\mathcal{R}_{\lambda^{[k]}}(\boldsymbol{\beta}_{01}^{[k]}) = \left(p'_{\lambda_1^{[k]}}\left(\left|\beta_{01}^{[k]}\right|\right)\mathrm{sign}\left(\beta_{01}^{[k]}\right),\ldots,p'_{\lambda_{q_k}^{[k]}}\left(\left|\beta_{0q_k}^{[k]}\right|\right)\mathrm{sign}\left(\beta_{0q_k}^{[k]}\right)\right)^{\mathrm{T}},$$

$$\Sigma_{\lambda^{[k]}}(\boldsymbol{\beta}_{01}^{[k]}) = \mathrm{diag}\left(p''_{\lambda_1^{[k]}}\left(\left|\beta_{01}^{[k]}\right|\right),\ldots,p''_{\lambda_{q_k}^{[k]}}\left(\left|\beta_{0q_k}^{[k]}\right|\right)\right),$$

$$\mathcal{B}_{[k]} = \mathrm{plim}_{N\to\infty}N^{-1}\sum_{i=1}^{N}\left[\left\{\eta'_k\left(X_{i[q_k]}^{\mathrm{T}}\boldsymbol{\beta}_{01}^{[k]}\right)\right\}^2\left\{X_{i[q_k]}-E\left(X_{i[q_k]}\middle|X_{i[q_k]}^{\mathrm{T}}\boldsymbol{\beta}_{01}^{[k]}\right)\right\}^{\otimes 2}\right],$$

$\sigma^2 = E\varepsilon^2$ *and "*$\mathrm{plim}_{N\to\infty}$*" stands for converges in probability.*

Before we prove the main result, we state two preliminary lemmas in the following.

Lemma 1 *[3] Under Assumption A,*

$$\sup_t|\hat{X}'_k(t)-X'_k(t)| = O_P(b_N), k=1,\ldots,p, \tag{S1}$$

where b_N *satisfies* $\frac{b_N}{N^{-1/4}a_N}\to 0$ *as* $N\to\infty$ *and* a_N *is a sequence approaching zero.*

Lemma 2 *Let* $E(T|W^{\mathrm{T}}\boldsymbol{\beta}=w^{\mathrm{T}}\boldsymbol{\beta})=m(w^{\mathrm{T}}\boldsymbol{\beta})$*. Suppose its derivatives up to the second order are bounded for all* $\boldsymbol{\beta}\in\mathfrak{H}_N$*, where* $\mathfrak{H}_N$ *is defined in Condition (A1), and that* $E|T|^r$ *exists for some* $r>3$*. Let* $(W_i^{\mathrm{T}},T_i)^{\mathrm{T}}$*,* $i=1,2,\ldots N$ *be an independent and identically distributed (i.i.d.) sample from* $(W^{\mathrm{T}},T)^{\mathrm{T}}$*. Let* $\tau_{N,h}=h^2+\sqrt{\log N/(Nh)}$*. If Assumption (A1)-(A3) holds, then*

$$\left|\frac{1}{N}\sum_{i=1}^{N}K_h(W_i^{\mathrm{T}}\boldsymbol{\beta}-w^{\mathrm{T}}\boldsymbol{\beta})\left(\frac{W_i^{\mathrm{T}}\boldsymbol{\beta}-w^{\mathrm{T}}\boldsymbol{\beta}}{h}\right)^d T_i - f_{\boldsymbol{\beta}}(w^{\mathrm{T}}\boldsymbol{\beta})m(w^{\mathrm{T}}\boldsymbol{\beta})\mu_d \right.$$
$$\left. -\{f_{\boldsymbol{\beta}}(w^{\mathrm{T}}\boldsymbol{\beta})m(w^{\mathrm{T}}\boldsymbol{\beta})\}'\mu_{d+1}h\right| = O(\tau_{N,h}), a.s. \tag{S2}$$

where $\mu_d=\int K(u)u^d dv$*, and* $d=0,1,\ldots$*,* $K_h(\cdot)=h^{-1}K(\cdot/h)$*.*

Proof. From Condition (A1), we know that $\boldsymbol{\beta}$ satisfies $\|\boldsymbol{\beta}-\boldsymbol{\beta}_0\|\le C_1 n^{-1/2+c_0}$. Thus, Lemma 2 can be proved by using similar arguments of Lemma 6.1 of [4].
Proof of Theorem 1. Let $\gamma_{Nk}=N^{-1/2}+a_{Nk}^*$, $\mathbf{v}_k=(v_{k1},\ldots,v_{kp})$ and $\|\mathbf{v}_k\|_2=C$ for some positive large constant C. Moreover,

$$a_{Nk}^* = \max_{1\le j\le p}\left\{p'_{\lambda_j^{[k]}}\left(\left|\beta_{0j}^{[k]}\right|\right),\beta_{0j}^{[k]}\neq 0\right\}, \tag{S3}$$

where $\beta_{0j}^{[k]}$ is the j-th element of $\boldsymbol{\beta}_0^{[k]}$ for $j=1,\ldots,p$. Let $\boldsymbol{\beta}_{0N}^{[k]}=\boldsymbol{\beta}_0^{[k]}+\gamma_{Nk}\mathbf{v}_k$ and

$$D_{kN,1} = \frac{1}{2}\sum_{i=1}^{N}\left\{Y_{ki}-\hat{\eta}_k\left(X_i^{\mathrm{T}}\boldsymbol{\beta}_{0N}^{[k]}\right)\right\}^2-\frac{1}{2}\sum_{i=1}^{N}\left\{Y_{ki}-\hat{\eta}_k\left(X_i^{\mathrm{T}}\boldsymbol{\beta}_{0}^{[k]}\right)\right\}^2,$$

$$D_{kN,2} = N\sum_{j=1}^{q_k}\left\{p_{\lambda_j^{[k]}}\left(\left|\beta_{0j}^{[k]}+\gamma_{Nk}v_{kj}\right|\right)-p_{\lambda_j^{[k]}}\left(\left|\beta_{0j}^{[k]}\right|\right)\right\}.$$

We aim to show that, for any given $\epsilon>0$, there exists a large constant C such that

$$P\left\{\inf_{\mathcal{V}^{[k]}}\mathcal{L}_P\left(\boldsymbol{\beta}_{0N}^{[k]}\right)\ge\mathcal{L}_P\left(\boldsymbol{\beta}_0^{[k]}\right)\right\}\ge 1-\epsilon, \tag{S4}$$

where $\mathcal{V}^{[k]} = \{\mathbf{v}_k : \|\mathbf{v}_k\| = C\}$ for some large positive constant C. If (S4) holds, we can conclude that the penalized minimizer $\hat{\boldsymbol{\beta}}_0^{[k]}$ for k-th module is $O_P(N^{-1/2})$. Note that $\mathcal{L}_P\left(\boldsymbol{\beta}_{0N}^{[k]}\right) - \mathcal{L}_P\left(\boldsymbol{\beta}_0^{[k]}\right) = D_{kN,1} + D_{kN,2}$. We analyze $D_{kN,1}$ and $D_{kN,2}$ to achieve (S4). We finish the proof in three steps.
Step A.1. By applying Proposition 1 (iii) in [5], we obtain that

$$\hat{\eta}_k\left(X_i^{\mathrm{T}}\boldsymbol{\beta}_0^{[k]}\right) - \hat{\eta}_k\left(X_i^{\mathrm{T}}\boldsymbol{\beta}_{0N}^{[k]}\right) = -\eta_k'\left(X_i^{\mathrm{T}}\boldsymbol{\beta}_0^{[k]}\right)\left[X_i^{\mathrm{T}} - E\left(X_i^{\mathrm{T}}|X_i^{\mathrm{T}}\boldsymbol{\beta}_0^{[k]}\right)\right]\mathbf{v}_k\gamma_{Nk} + O_P(\gamma_{Nk}\mathbf{v}_k\tilde{\tau}_{N,h}), \quad \text{(S5)}$$

where $\tilde{\tau}_{N,h} = h^2 + \sqrt{1/(Nh^3)}$. Thus,

$$\begin{aligned} D_{kN,1} &= \frac{1}{2}\sum_{i=1}^N\left\{\hat{\eta}_k\left(X_i^{\mathrm{T}}\boldsymbol{\beta}_0^{[k]}\right) - \hat{\eta}_k\left(X_i^{\mathrm{T}}\boldsymbol{\beta}_{0N}^{[k]}\right)\right\}^2 + \frac{1}{2}\sum_{i=1}^N\left\{\hat{\eta}_k\left(X_i^{\mathrm{T}}\boldsymbol{\beta}_0^{[k]}\right) - \hat{\eta}_k\left(X_i^{\mathrm{T}}\boldsymbol{\beta}_{0N}^{[k]}\right)\right\} \\ &\quad \times\left\{2Y_{ki} - 2\eta_k\left(X_i^{\mathrm{T}}\boldsymbol{\beta}_0^{[k]}\right)\right\} + \frac{1}{2}\sum_{i=1}^N\left\{\hat{\eta}_k\left(X_i^{\mathrm{T}}\boldsymbol{\beta}_0^{[k]}\right) - \hat{\eta}_k\left(X_i^{\mathrm{T}}\boldsymbol{\beta}_{0N}^{[k]}\right)\right\} \\ &\quad \times\left\{2\eta_k\left(X_i^{\mathrm{T}}\boldsymbol{\beta}_0^{[k]}\right) - 2\hat{\eta}_k\left(X_i^{\mathrm{T}}\boldsymbol{\beta}_0^{[k]}\right)\right\} \stackrel{\text{def}}{=} D_{kN,1}^{(1)} + D_{kN,1}^{(2)} + D_{kN,1}^{(3)}. \end{aligned}$$

Using (S5) and recalling that $\gamma_{Nk} = N^{-1/2} + a_{kN}^*$, we have $N\gamma_{Nk}^2 = O(1)$. Then

$$\begin{aligned} D_{kN,1}^{(1)} &= \frac{\gamma_{Nk}^2}{2}\sum_{i=1}^N\left[\eta_k'\left(X_i^{\mathrm{T}}\boldsymbol{\beta}_0^{[k]}\right)\right]^2\mathbf{v}_k^{\mathrm{T}}\left[X_i - E\left(X_i|X_i^{\mathrm{T}}\boldsymbol{\beta}_0^{[k]}\right)\right]^{\otimes 2}\mathbf{v}_k(1 + o_P(1)) \\ &= N\gamma_{Nk}^2\mathbf{v}_k^{\mathrm{T}}\mathbf{A}\mathbf{v}_k(1 + o_P(1)) \geq C^2\lambda_{\min}(\mathbf{A})(1 + o_P(1)), \end{aligned} \quad \text{(S6)}$$

where $\mathbf{A} = \operatorname{plim}_{N\to\infty}N^{-1}\sum_{i=1}^N\left\{\left[\eta_k'\left(X_i^{\mathrm{T}}\boldsymbol{\beta}_0^{[k]}\right)\right]^2\left[X_i - E\left(X_i|X_i^{\mathrm{T}}\boldsymbol{\beta}_0^{[k]}\right)\right]^{\otimes 2}\right\}$, and $\lambda_{\min}(\mathbf{A})$ is the smallest eigenvalue of $\mathbf{A}$. Lemma 1 entails that $Y_{ki} = M_k(t_i) + O_P(b_N)$. Moreover, using Lemma 2 and the argument similar to Theorem 3 in [6], we have $\hat{\eta}_k\left(X_i^{\mathrm{T}}\boldsymbol{\beta}_0^{[k]}\right) - \eta_k\left(X_i^{\mathrm{T}}\boldsymbol{\beta}_0^{[k]}\right) = O_P(\tau_{N,h} + b_N)$. Thus, together with (S5), we can have

$$\begin{aligned} D_{NK,1}^{(2)} &= \sum_{i=1}^N\left\{\eta_k'\left(X_i^{\mathrm{T}}\boldsymbol{\beta}_0^{[k]}\right)\left[X_i^{\mathrm{T}} - E\left(X_i|X_i^{\mathrm{T}}\boldsymbol{\beta}_0^{[k]}\right)\right]\mathbf{v}_k\gamma_{Nk} + \mathbf{v}_kO_P(\gamma_{Nk}\tilde{\tau}_{n,h})\right\}\{\varepsilon_i + O_P(b_N)\} \\ &= \mathbf{v}_k[N^{1/2}\gamma_{Nk}\{O_P(1) + O_P(b_N) + O_P(\tilde{\tau}_{n,h})\} + O_P(N\gamma_{Nk}b_N\tilde{\tau}_{n,h})] \\ &= \mathbf{v}_k[O_P(1) + o_P(1)] = C \times O_P(1), \end{aligned} \quad \text{(S7)}$$

$$\begin{aligned} D_{NK,1}^{(3)} &= \sum_{i=1}^N\left\{\eta_k'\left(X_i^{\mathrm{T}}\boldsymbol{\beta}_0^{[k]}\right)\left[X_i^{\mathrm{T}} - E\left(X_i|X_i^{\mathrm{T}}\boldsymbol{\beta}_0^{[k]}\right)\right]\mathbf{v}_k\gamma_{Nk} + \mathbf{v}_kO_P(\gamma_{Nk}\tilde{\tau}_{N,h})\right\}O_P(\tau_{N,h} + b_N) \\ &= \mathbf{v}_k[N^{1/2}\gamma_{Nk}O_P(\tau_{N,h} + b_N) + O_P(N\gamma_{Nk}\tilde{\tau}_{N,h})O_P(\tau_{N,h} + b_N)] \\ &= \mathbf{v}_ko_P(1) = C \times o_P(1). \end{aligned} \quad \text{(S8)}$$

In the last equalities of (S7)-(S8), we invoked that $h^3\log N \to 0$, $Nh^8 \to 0$, $\frac{\log N}{Nh^4} \to 0$ and $\frac{a_N^4}{Nh^6} \to 0$. These bandwidth conditions yielded Condition (A3). Consequently, we obtain that $D_{Nk,1} \geq C^2\lambda_{\min}(\mathbf{A})(1 + o_P(1)) + C \times [O_P(1) + o_P(1)]$.
Step A.2. Taylor expansion and the Cauchy-Schwarz inequality entail that

$$\begin{aligned} |D_{Nk,2}| &\leq \sqrt{q_k}N\gamma_{Nk}a_{Nk}^*\|\mathbf{v}_k\|_2 + N\gamma_{Nk}^2b_{Nk}^*\|\mathbf{v}_k\|_2^2 \\ &= C \times O(1) + C^2b_{Nk}^* = C^2o(1) + C \times O(1), \end{aligned} \quad \text{(S9)}$$

where $b^*_{Nk} = \max_{1\le j\le p}\left\{p''_{\lambda_j^{[k]}}\left(\left|\beta_{0j}^{[k]}\right|\right), \beta_{0j}\neq 0\right\}$. Together with the results of Step A.1, when b^*_{Nk} tends to 0 and C is sufficiently large, $D_{Nk,1}$ dominates the $D_{Nk,2}$. As a result, for any given $\epsilon>0$, there exists a large constant C such that (S4) holds.

Let $\boldsymbol{\beta}_1^{[k]}$ satisfy $\|\boldsymbol{\beta}_1^{[k]}-\boldsymbol{\beta}_{01}^{[k]}\| = O_P(N^{-1/2})$. We now show that

$$\mathcal{L}_P\left\{\left(\begin{array}{c}\boldsymbol{\beta}_1^{[k]}\\ \mathbf{0}\end{array}\right)\right\} = \min_{\mathcal{C}^{[k]}}\mathcal{L}_P\left\{\left(\begin{array}{c}\boldsymbol{\beta}_1^{[k]}\\ \boldsymbol{\beta}_2^{[k]}\end{array}\right)\right\}, \tag{S10}$$

where $\mathcal{C}^{[k]} = \left\{\left\|\boldsymbol{\beta}_2^{[k]}\right\|\le C^*N^{-1/2}\right\}$ for some positive constant C^*. Consider $\beta_j^{[k]}$, $j=q_k+1,\ldots,p$, when $\beta_j^{[k]}\neq 0$, $\frac{\partial\mathcal{L}_P(\boldsymbol{\beta}^{[k]})}{\partial\beta_j^{[k]}} = -\sum_{i=1}^N\left\{Y_i-\hat{\eta}_k\left(X_i^{\mathrm{T}}\boldsymbol{\beta}^{[k]}\right)\right\}\frac{\partial\hat{\eta}_k(X_i^{\mathrm{T}}\boldsymbol{\beta}^{[k]})}{\partial\beta_j^{[k]}} + Np'_{\lambda_j^{[k]}}\left(\left|\beta_j^{[k]}\right|\right)\mathrm{sign}\left(\beta_j^{[k]}\right)$. Using Proposition 1 (iii) in [5] or Lemma 1 in [7], we can have

$$\begin{aligned}
\frac{\partial\mathcal{L}_P(\boldsymbol{\beta}^{[k]})}{\partial\beta_j^{[k]}} &= Np'_{\lambda_j^{[k]}}\left(\left|\beta_j^{[k]}\right|\right)\mathrm{sign}\left(\beta_j^{[k]}\right) - \sum_{i=1}^N\left\{Y_i-\hat{\eta}_k\left(X_i^{\mathrm{T}}\boldsymbol{\beta}^{[k]}\right)\right\}\eta'_k\left(X_i^{\mathrm{T}}\boldsymbol{\beta}_0^{[k]}\right)\\
&\times\left[\left\{X_{ij}-E\left(X_{ij}|X_i^{\mathrm{T}}\boldsymbol{\beta}_0^{[k]}\right)\right\} - \frac{\beta_{0j}^{[k]}\{X_{i1}-E(X_{i1}|X_i^{\mathrm{T}}\boldsymbol{\beta}_0^{[k]})\}}{\sqrt{1-\|\boldsymbol{\beta}_{0_{-1}}^{[k]\mathrm{T}}\boldsymbol{\beta}_{0_{-1}}^{[k]}\|_2^2}} + O_P(N^{-1/2}+\tilde{\tau}_{N,h})\right]\\
&\stackrel{\text{def}}{=} \Lambda_{N1}-\Lambda_{N2},
\end{aligned}$$

where $\boldsymbol{\beta}_{0_{-1}}^{[k]} = \left(\beta_{02}^{[k]},\ldots,\beta_{0p}^{[k]}\right)^{\mathrm{T}}$. For Λ_{N2}, using that $Y_i-\hat{\eta}_k\left(X_i^{\mathrm{T}}\boldsymbol{\beta}^{[k]}\right) = \varepsilon_i+\eta_k\left(X_i^{\mathrm{T}}\boldsymbol{\beta}_0^{[k]}\right)-\hat{\eta}_k\left(X_i^{\mathrm{T}}\boldsymbol{\beta}^{[k]}\right)+O_P(b_N)$, and applying the similar arguments of Step II in the proof of Theorem 2 in [8], we have $\Lambda_{N2} = O_P(N^{1/2})\times[O_P(1)+o_P(1)+O_P(N^{1/2}\tau_{N,h}\tilde{\tau}_{N,h})+O_P(N^{1/2}b_N\tilde{\tau}_{N,h})] = O_P(N^{1/2})$ as $h^3\log N\to 0$, $Nh^8\to 0$, $\frac{\log N}{Nh^4}\to 0$ and $\frac{a_N^4}{Nh^6}\to 0$. Using Condition (A7), we further have that

$$\begin{aligned}
\frac{\partial\mathcal{L}_P(\boldsymbol{\beta}^{[k]})}{\partial\beta_j^{[k]}} = \Lambda_{N1}-\Lambda_{N2} &= N\lambda_j^{[k]}\left[\mathrm{sign}\left(\beta_j^{[k]}\right)p'_{\lambda_j^{[k]}}\left(\left|\beta_j^{[k]}\right|\right)/\lambda_j^{[k]} + O_P\left(N^{-1/2}/\lambda_j^{[k]}\right)\right]\\
&= N\lambda_j^{[k]}\left[\mathrm{sign}\left(\beta_j^{[k]}\right)p'_{\lambda_j^{[k]}}\left(\left|\beta_j^{[k]}\right|\right)/\lambda_j^{[k]} + o_P(1)\right].
\end{aligned} \tag{S11}$$

This indicates that $\frac{\partial\mathcal{L}_P(\boldsymbol{\beta}^{[k]})}{\partial\beta_j^{[k]}}$ has different signs for $\beta_j^{[k]}\in(-C^*N^{-1/2}, C^*N^{-1/2})$. As a consequence, the minimum is only attained at $\beta_j^{[k]}=0$ for $j=q_k+1,\ldots,p$. We complete the proof of (S10).

Step A.3. From Step A.2, we know that $\hat{\boldsymbol{\beta}}_{02}^{[k]} = \mathbf{0}_{p-q_k}$. Similar to theses arguments used by [8], as $h^3\log N\to 0$, $Nh^8\to 0$, $\frac{\log N}{Nh^4}\to 0$ and $\frac{a_N^4}{Nh^6}\to 0$, it follows that the penalized estimator $\hat{\boldsymbol{\beta}}_{01}^{[k]}$ satisfies

$$\begin{aligned}
\mathbf{0}_{q_k\times 1} &= -\sum_{i=1}^N\left[Y_i-\hat{\eta}_k\left(X_{i[q_k]}^{\mathrm{T}}\hat{\boldsymbol{\beta}}_{01}^{[k]}\right)\right]\eta'_k\left(X_{i[q_k]}^{\mathrm{T}}\hat{\boldsymbol{\beta}}_{01}^{[k]}\right)\left[X_{i[q_k]}-E\left(X_{i[q_k]}|X_{i[q_k]}^{\mathrm{T}}\boldsymbol{\beta}_{01}^{[k]}\right)\right]\\
&\quad +N\mathcal{R}_{\lambda^{[k]}}\left(\hat{\boldsymbol{\beta}}_{01}^{[k]}\right)+o_P(N^{1/2}),
\end{aligned} \tag{S12}$$

where $\mathcal{R}_{\lambda^{[k]}}\left(\hat{\boldsymbol{\beta}}_{01}^{[k]}\right) = \left(p'_{\lambda_1^{[k]}}\left(\left|\hat{\beta}_{01}^{[k]}\right|\right)\mathrm{sign}\left(\hat{\beta}_{01}^{[k]}\right),\ldots,p'_{\lambda_{q_k}^{[k]}}\left(\left|\hat{\beta}_{0q_k}^{[k]}\right|\right)\mathrm{sign}\left(\hat{\beta}_{0q_k}^{[k]}\right)\right)^{\mathrm{T}}$. Furthermore, we define

$$\mathcal{R}_{\lambda^{[k]}}\left(\boldsymbol{\beta}_{01}^{[k]}\right) = \left(p'_{\lambda_1^{[k]}}\left(\left|\beta_{01}^{[k]}\right|\right)\mathrm{sign}\left(\beta_{01}^{[k]}\right),\ldots,p'_{\lambda_{q_k}^{[k]}}\left(\left|\beta_{0q_k}^{[k]}\right|\right)\mathrm{sign}\left(\beta_{0q_k}^{[k]}\right)\right)^{\mathrm{T}},$$

$$\Sigma_{\lambda^{[k]}}\left(\boldsymbol{\beta}_{01}^{[k]}\right) = \operatorname{diag}\left(p''_{\lambda_1^{[k]}}\left(\left|\beta_{01}^{[k]}\right|\right),\ldots,p''_{\lambda_{q_k}^{[k]}}\left(\left|\beta_{0q_k}^{[k]}\right|\right)\right).$$

Applying Taylor expansion to (S12) with respect to $\hat{\boldsymbol{\beta}}_{01}^{[k]}$ around $\boldsymbol{\beta}_{01}^{[k]}$, we have

$$\begin{aligned}\frac{1}{\sqrt{N}}\sum_{i=1}^{N}\varepsilon_i\eta'_k(X_{i[q_k]}^{\mathrm{T}}\boldsymbol{\beta}_{01}^{[k]})\left[X_{i[q_k]}-E\left(X_{i[q_k]}|X_{i[q_k]}^{\mathrm{T}}\boldsymbol{\beta}_{01}^{[k]}\right)\right] &= \mathcal{B}_{N,\lambda^{[k]}}\sqrt{N}\left(\hat{\boldsymbol{\beta}}_{01}^{[k]}-\boldsymbol{\beta}_{01}^{[k]}\right)\\ &\quad +N^{1/2}\mathcal{R}_{\lambda^{[k]}}\left(\boldsymbol{\beta}_{01}^{[k]}\right)+o_P(1), \quad \text{(S13)}\end{aligned}$$

where

$$\begin{aligned}\mathcal{B}_{N,\lambda^{[k]}} &= \frac{1}{N}\sum_{i=1}^{N}\left\{\eta'_k\left(X_{i[q_k]}^{\mathrm{T}}\boldsymbol{\beta}_{01}^{[k]}\right)\right\}^2\left\{X_{i[q_k]}-E\left(X_{i[q_k]}|X_{i[q_k]}^{\mathrm{T}}\boldsymbol{\beta}_{01}^{[k]}\right)\right\}^{\otimes 2}+\Sigma_{\lambda^{[k]}}\left(\boldsymbol{\beta}_{01}^{[k]}\right)\\ &\xrightarrow{P} \lim_{N\to\infty}N^{-1}\sum_{i=1}^{N}\left[\left\{\eta'_k\left(X_{i[q_k]}^{\mathrm{T}}\boldsymbol{\beta}_{01}^{[k]}\right)\right\}^2\left\{X_{i[q_k]}-E\left(X_{i[q_k]}|X_{i[q_k]}^{\mathrm{T}}\boldsymbol{\beta}_{01}^{[k]}\right)\right\}^{\otimes 2}\right]+\Sigma_{\lambda^{[k]}}(\boldsymbol{\beta}_{01}^{[k]})\\ &\stackrel{\text{def}}{=} \mathcal{B}_{[k]}+\Sigma_{\lambda^{[k]}}(\boldsymbol{\beta}_{01}^{[k]}). \quad \text{(S14)}\end{aligned}$$

(S13)-(S14) indicate that

$$\begin{aligned}&\sqrt{N}\left[\left(\hat{\boldsymbol{\beta}}_{01}^{[k]}-\boldsymbol{\beta}_{01}^{[k]}\right)+\left(\mathcal{B}_{[k]}+\Sigma_{\lambda^{[k]}}\left(\boldsymbol{\beta}_{01}^{[k]}\right)\right)^{-1}\mathcal{R}_{\lambda^{[k]}}\left(\boldsymbol{\beta}_{01}^{[k]}\right)\right]\\ &=\left(\mathcal{B}_{[k]}+\Sigma_{\lambda^{[k]}}\left(\boldsymbol{\beta}_{01}^{[k]}\right)\right)^{-1}\frac{1}{\sqrt{N}}\sum_{i=1}^{N}\varepsilon_i\eta'_k\left(X_{i[q_k]}^{\mathrm{T}}\boldsymbol{\beta}_{01}^{[k]}\right)\left[X_{i[q_k]}-E\left(X_{i[q_k]}|X_{i[q_k]}^{\mathrm{T}}\boldsymbol{\beta}_{01}^{[k]}\right)\right]+o_P(1)\\ &\xrightarrow{L} N\left(\mathbf{0}_{q_k\times 1},\sigma^2\left(\mathcal{B}_{[k]}+\Sigma_{\lambda^{[k]}}\left(\boldsymbol{\beta}_{01}^{[k]}\right)\right)^{-1}\mathcal{B}_{[k]}\left(\mathcal{B}_{[k]}+\Sigma_{\lambda^{[k]}}\left(\boldsymbol{\beta}_{01}^{[k]}\right)\right)^{-1}\right).\end{aligned}$$

We complete the proof.

Computational cost

[9] pointed the computational complexity of SSC approximately $O(n^2N^2)$, where n denoted the number of genes. Hence, Step 1 takes $O(n^2N^2Kn) = O(n^3N^2K)$ time to classify genes into modules with the maximum cluster number K. Besides, traditional computational methods for LME combines Expectation-Maximization (EM) and Newton-Raphson (NR) algorithm. According to [**?**], the computational complexity to estimate $\mathbf{X}(\mathbf{t})$ for module i is $O(s_{1i}N^3n_i^3 + s_{2i}N^3n_i^7)$, where s_{1i} and s_{2i} denote the maximum numbers of iterations of EM and NR algorithms. Then it takes approximately $O(pq^2)$ (q is the order of spline function) time to estimate the first derivative of $\widehat{X}(t)$. Therefore the total computational complexity of Step 2 is $O(\sum_{i=1}^{p}(s_{1i}N^3n_i^3 + s_{2i}N^3n_i^7) + pq^2)$. Referred to [8], Step 3 consumes time approximately $O(n_\lambda ps_3(N^2p + s_4Np)) = O(n_\lambda p^2Ns_3(N + s_4))$, where n_λ is the range size of $\lambda^{[k]}$, and s_3 and s_4 denote the maximum iterated number of PPrLS and SCAD approaches, respectively. In summary all three steps' lower bound of the computational complexity are $O(n^3)$, $O(\sum_{i=1}^{p} n_i^7)$ and $O(p^2N^2)$. It's worth noting that Steps 1 and 2 domain the overall complexity, and cluster number (p) and size (n_i) control the computational efficiency of Step 2.

References

1. Lu T, Liang H, Li H, Wu H. High-dimensional ODEs coupled with mixed-effects modeling techniques for dynamic gene regulatory network identification. Journal of the American Statistical Association. 2011;106:1242–1258.

2. Fan J, Li R. Variable Selection via Nonconcave Penalized Likelihood and its Oracle Properties. Journal of the American Statistical Association. 2001;96:1348–1360.

3. Wu H, Zhang JT. Nonparametric Regression Methods for Longitudinal Data Analysis. Wiley Series in Probability and Statistics. Hoboken, NJ: Wiley-Interscience; 2006.

4. Xia Y. Asymptotic distributions for two estimators of the single-index model. Econometric Theory. 2006;22:1112–1137.

5. Cui X, Härdle W, Zhu LX. The EFM approach for single-index models. The Annals of Statistics. 2011;39:1658–1688.

6. Fan J, Gijbels I. Local Polynomial Modelling and Its Applications. vol. 66. London: Chapman & Hall; 1996.

7. Zhang J, Wu P, Gai Y. Estimation in linear regression models with measurement errors subject to single-indexed distortion. Computational Statistics & Data Analysis. 2012;59:103–120.

8. Liang H, Liu X, Li R, Tsai CL. Estimation and testing for partially linear single-index models. The Annals of Statistics. 2010;38:3811–3836.

9. Ma P, Castillo-Davis CI, Zhong W, Liu JS. A data-driven clustering method for time course gene expression data. Nucleic Acids Research. 2006;34:1261–1269.
